# The beetle fauna (Insecta, Coleoptera) of the Rawdhat Khorim National Park, Central Saudi Arabia

**DOI:** 10.3897/zookeys.653.10252

**Published:** 2017-02-07

**Authors:** Mahmoud S. Abdel-Dayem, Hassan H. Fad, Ashraf M. El-Torkey, Ali A. Elgharbawy, Yousif N. Aldryhim, Boris C. Kondratieff, Amin N. Al Ansi, Hathal M. Aldhafer

**Affiliations:** 1 King Saud University Museum of Arthropods (KSMA), Plant Protection Department, College of Food and Agriculture Sciences, King Saud University, P.O. Box 2460 Riyadh 11451, Saudi Arabia; 2 Entomology Department, Faculty of Science, Ain Shams University, Cairo, Egypt; 3 Plant Protection Research Institute, Agriculture Research Center, Giza, Egypt; 4 Zoology Department, Faculty of Science, Al Azhar University, Nasr City, Cairo, Egypt; 5 Department of Bioagricultural Sciences and Pest Management, Colorado State University, Campus Delivery 1177, Fort Collins, Colorado, U.S.A. 80523

**Keywords:** Arabian fauna, distribution, endemic species, new records, zoogeography

## Abstract

This study was conducted as a part of a comprehensive baseline survey of insect biodiversity of Rawdhat Khorim National Park (RKNP), Central Kingdom of Saudi Arabia (KSA). During this study a total of 262 Coleoptera species belong to 182 genera in 35 families were identified, of which 247 are named at a species level. Fifteen species (6.0%) are apparently endemic to KSA. Thirty-eight species are new to the known beetle fauna of KSA, including 25 species reported from the Arabian Peninsula for the first time. The families Tenebrionidae (45 species), Scarabaeidae (34 species), and Carabidae (27 species) were the most species rich families. About 37% of the beetle abundance was represented by species of Scarabaeidae, especially *Aphodius ictericus ghardimaouensis* Balthasar. *Karumia inaequalis* Pic (Dascillidae) was also an abundant species. Approximately 43.5% of beetle species collected during this study are considered very rare taxa in RKNP. The RKNP beetle fauna shows more affinity to Sahro-Arabian (36.4%), Afrotropical-Sahro-Arabian (17.4%) and Palaearctic-Sahro-Arabian (10.5%). Twenty-three species (9.3%) are considered cosmopolitan or subcosmopolitan. The data on month of collection, method of collection, and abundance status within RKNP, together with the distribution within KSA and the general distribution (zoogeography) of each species are presented.

## Introduction

Beetles (Coleoptera) are considered the most taxonomically diverse insect group that comprises major components of ecosystems in terms of biomass, species richness and ecological roles (Stack 2015). About 400,000 species have been described (Hammond 1992), comprising about 25% of the Earth’s total animal diversity (Rosenzweig 1995; Hunt et al. 2007). Beetles play important roles in pollination, herbivory, granivory, predator-prey interactions, decomposition and nutrient cycling, and soil disturbances (Huffaker and Gutierrez 1999).

The foundation of our knowledge of the beetles of the Kingdom of Saudi Arabia (KSA) was presented by various authors in the series “Fauna of Saudi Arabia,” currently published as part of the “Fauna of Arabia” (Wittmer and Buttiker 1979- to date). Additionally, sixteen years ago, Al-Ahmadi and Salem (1999) listed 1,343 beetle species from KSA. Additional data on the beetles in KSA are available in the Catalogue of Palaearctic Coleoptera (Löbl and Smetana 2003–2007).

Rawdhats, naturally occurring moist basins, are one of the major components that considered as biological cores in the hyper-arid ecosystems of Central KSA (Tag El-Din et al. 1994; Al-Farraj et al. 1997; Alfarhan 2001; Al-Qarawi 2011). The flora and fauna of these unique habitats are under constant threat due to increased anthropogenic activities (Al-Nafie 2007; Al-Qarawi 2011). However, relatively little is known about their insect fauna (e.g., Al Dhafer and Platia 2013, Al Dhafer et al. 2016; Sharaf et al. 2013; Alqarni et al. 2015; Abdel-Dayem et al. 2015, 2016) that contribute to the faunal biodiversity of KSA.

Rawdhat Khorim National Park (RKNP) in Riyadh Province is important element in the conservation network of KSA and its landscape is characterized by relative diverse pristine native plant communities. (Tag El-Din et al. 1994; Al-Farraj et al. 1997; Alfarhan 2001; Al-Qarawi 2011). Recent studies of the insects occurring in RKNP have revealed several new taxa, Coleoptera: *Dicronychus latifahae* (Elateridae) (Al Dhafer and Platia 2013); *Reichardtiolus aldhaferi* (Histeridae) (Lackner 2014); *Boromorphus saudicus* (Tenebrionidae) (Schawaller et al. 2013); and Hymenoptera: *Tetramorium saudicum* (Formicidae) (Sharaf et al. 2013).

The objective of the current study was to provide a comprehensive list of the beetles from one of the more unique habitats in the world, specifically RKNP contributing to the knowledge of the beetle fauna of KSA and the Arabian Peninsula in general.

## Materials and methods

### Study area

**Figure 1.**
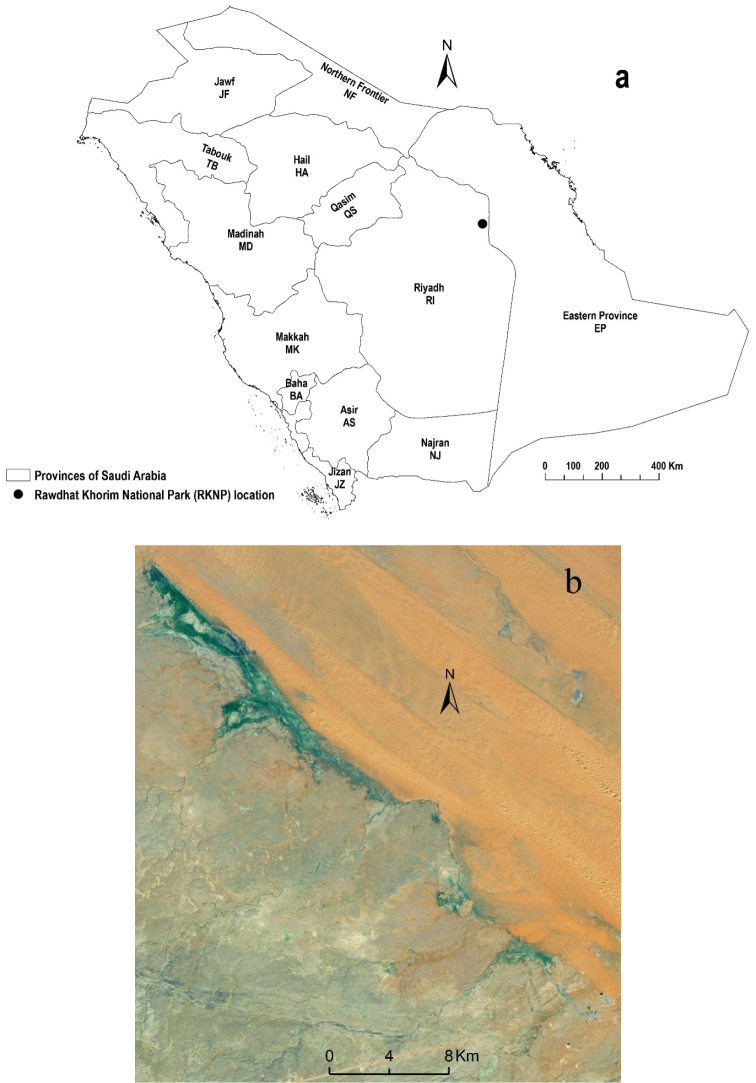
**a** Map showing the provinces and geographic location of Rawdhat Khorim National Park within the Central Region of Kingdom of Saudi Arabia **b** Photo of Rawdhat Khorim National Park (Produced by ArcMap 10.4).

The Rawdhat Khorim National Park is situated in the northeastern Riyadh Province of Central KSA (Fig. 1a). This is part of the Najd Plateau, a sedimentary rectangular plateau of Saudi Arabia. It covers an area of 24 km^2^ and located about 95 km northeast of the capital of Riyadh (25°23’N, 47°17’E, 560 m.a.s.l.). It is a densely vegetated alluvial basin (Vesey-Fizberald 1957) (Figure 1b) supporting a characteristic floral community in the hyper-arid desert of central Saudi Arabia (Al-Farraj et al. 1997; Alfarhan 2001). This area has low rainfall, but drainage from surrounding foothills of the adjacent highlands provides additional moisture forming temporary water pools and high plant diversity occurs during spring (Al-Farraj et al. 1997).

### Climate

The climate of RKNP is characterized by a hot summer and a mild winter with an average relative humidity of 33%. The average annual temperature is 26°C, June to August is the hottest period of the year (35-37.4 °C) and December to February is the coldest (14.6-15.7 °C). The average annual precipitation is 122 mm, the highest amount of precipitation occurs during March and April (30 and 27 mm, respectively), while no rainfall occurs during June to September (Sharaf et al. 2013).

### Flora

The flora of RKNP includes a total of 153 plant species belonging to 32 families (Alfarhan 2001). The vegetation is complex consisting of perennial and annual herbs, shrubs and trees (Al-Farraj et al. 1997). *Matricaria aurea* (Loefl.) Sch.-Bip. (Compositae)), *Plantago boissieri* Hausskn. & Bornm., *Plantago ciliata* Desf. (Plantaginaceae), and *Phalaris minor* Retz. (Poaceae) are the dominant annual herbs (Al-Farraj et al. 1997; Alfarhan 2001). *Acacia ehrenbergiana* Heyne, *Acacia gerrardii* Benth. (Fabaceae), *Calotropis procera* (Aiton) W.T. Aiton (Apocynaceae), *Lycium shawii* Roem. & Schult. (Solanaceae), *Rhazya stricta* Decne. (Apocynaceae) and *Ziziphus nummularia* (Burm. f) Wight & Arn. (Rhamnaceae) are the dominated shrub and trees (Al-Farraj et al. 1997). Plant growth is higher during spring than in other seasons (Al-Farraj et al. 1997).

### Beetle collection

The species listed here are based on the survey that was conducted biweekly from October, 2011 to September, 2013 in the RKNP. The material was collected using a variety of collecting methods including pitfall trapping (PT), UV-light trapping (LT), Malaise trapping (MT), net sweeping (SW), beating vegetation (BV), vacuuming (VC), and hand-picking (HP). All collected beetles were sorted, identified and deposited in the King Saud University Museum of Arthropods (KSMA), College of Food and Agricultural Sciences, King Saud University, KSA.

### Species format and arrangement

**Figure 2.**
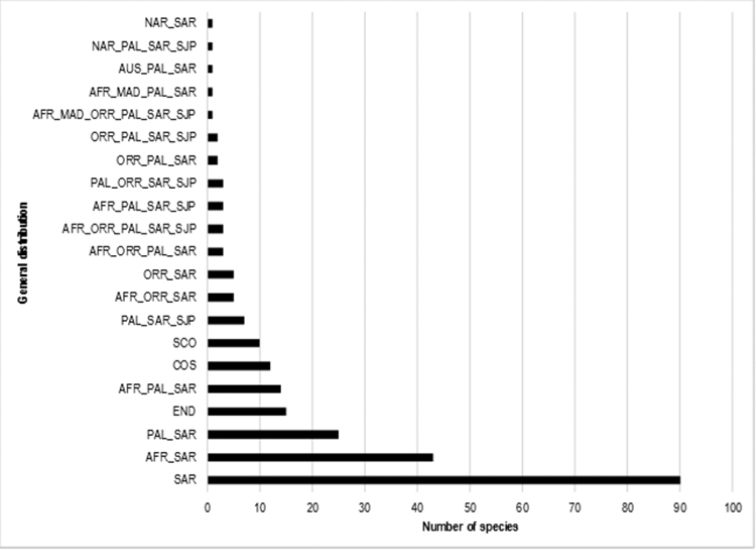
The general distributions frequency of the beetle fauna of Rawdhat Khorim National Park, Central Kingdom of Saudi Arabia.

Species identification is based on specialists “see Acknowledgments” and numerous publications not included here. Beetles were not being identified to the species level were included in the study if specimens were morphologically different from other (congeneric) species. The identified species are arranged systematically to subfamily level and alphabetically thereafter. The classification and nomenclature of subfamilies and higher levels follows Bouchard et al. (2011). The valid name followed by the author and date of publication for species were given and these were not cited in the reference section and they can be found in the Catalogue of Palaearctic Coleoptera (Löbl and Smetana 2003–2007). The world distribution for each species is indicated as two capital letters for each country (according ISO 3166: http://www.iso.org/iso/country_codes). The world distribution is based mainly on Catalogue of Palaearctic Coleoptera (Löbl and Smetana 2003–2007), Global Biodiversity Information Facility (GBIF: http://www.gbif.org), and Beetles and Rock Art in Libya (http://jcringenbach.free.fr). The general distribution (zoogeography) of each species is also indicated as a letter code (see “Abbreviations”) corresponding to main zoogeographic regions of the world proposed by Holt et al. (2013) (Fig. 2). The KSA distribution of each species is indicated (abbreviated as two letters for the Saudi provinces) (Fig. 1a). The KSA distributional records are based mainly on published records in the series “Fauna of Saudi Arabia”, being published as part of the “Fauna of Arabia (Wittmer and Buttiker 1979-to date) and other available papers on Saudi beetles. The absence of a geographic entry after a species name indicates that the species was recorded from Arabia or KSA but no locality was specified. For each species the following information is given: local abundance (all values of collected specimens were log transformed and then they classified into five categories using equal interval classification: very rare: <5 individuals, rare: 5-17, frequent: 18-70, common: 71-300, and abundant: > 300); collecting method (see abbreviation in “Beetle collection” above); and months of collection (Roman numerals) within RKNP.

### Abbreviations for the zoogeographical regions

AFR
 Afrotropical 


AUS
 Australian 


COS
 Cosmopolitan 


MAD
 Madagascan 


NAR
 Nearctic 


NTR
 Neotropical 


OCN
 Oceanic 


ORR
 Oriental 


PAL
 Palaearctic 


SAR
 Saharo_Arabian 


SCO
 Subcosmopolitan 


SJP
 Sino_Japanese 


## Results and discussion

This study represents the first inventory of beetles in RKNP, within arid region of central KSA. In total, 15,658 specimens were collected and identified to 262 species belonging to 182 genera included in 83 subfamilies and 35 families. Forty percent of known faunal diversity is accounted for by three beetle families, Tenebrionidae (45 spp.), Scarabaeidae (34 spp.) and Carabidae (27 spp.) (Table 1). The families with the greatest abundant were Scarabaeidae (36.6%) followed by Tenebrionidae (16.7%) (Table 1). Thirty-eight species have not been previously reported from KSA are listed, including 25 new records for the Arabian Peninsula. Ten families were represented by single species in the RKNP.

**Table 1.** The taxonomical and faunistic analysis of the beetle fauna in Rawdhat Khorim National Park, Central Kingdom of Saudi Arabia.

Family	Subfamily	Genera	Species	New record	Relative abundance
Anthicidae	2	5	7	1	0.27
Bolboceratidae	1	1	1	0	0.01
Bostrichidae	2	3	3	1	1.30
Brentidae	2	2	2	0	1.10
Buprestidae	5	7	10	0	1.30
Carabidae	8	22	27	8	5.36
Cerambycidae	2	2	2	0	0.11
Chrysomelidae	5	8	8	1	3.95
Cleridae	2	4	4	0	0.51
Coccinellidae	1	8	15	0	3.44
Cryptophagidae	2	2	2	1	0.14
Curculionidae	4	15	20	0	1.99
Dascillidae	1	1	1	0	8.02
Dermestidae	4	6	15	5	1.58
Dytiscidae	2	3	3	0	0.56
Elateridae	2	8	13	2	9.30
Glaresidae	0	1	2	0	0.02
Heteroceridae	1	1	2	2	0.08
Histeridae	3	8	14	0	0.54
Hybosoridae	1	1	1	0	3.48
Hydrophilidae	1	1	1	1	0.04
Laemophloeidae	1	1	1	1	0.01
Leiodidae	1	1	1	1	0.01
Meloidae	2	2	4	0	0.17
Melyridae	2	3	4	1	1.23
Mycetophagidae	1	1	1	0	0.02
Nitidulidae	3	3	3	0	0.70
Oedemeridae	1	1	1	0	0.03
Phalacridae	1	1	1	0	0.17
Ptinidae	2	2	3	2	0.06
Scarabaeidae	7	18	34	3	36.62
Scraptiidae	1	1	2	0	0.47
Staphylinidae	5	7	8	4	0.66
Tenebrionidae	4	31	45	4	16.71
Thanerocleridae	1	1	1	0	0.03
**Total 35**	**83**	**182**	**262**	**38**	

Eleven species (4.2%) are classified as abundant species, from which *Aphodius ictericus ghardimaouensis* (Scarabaeidae) (1,238 specimens) and *Karumia inaequalis* (Dascillidae) (1,262 specimens) were the highest abundant species. Thirty-six (13.7%) and 46 (17.6%) species are concidered as common and frequent, respectively. While, 64.5% of the species considered as rare (55 spp.) or very rare (114 spp.).

The RKNP beetle fauna demonstrated variable zoogeographical affinities (Figure 2), 15 species (6.1%) are endemic to KSA. The remainder of the species showed high affinities to SAR (36.4%), AFR_SAR (17.4%), and PAL_SAR (10.5%).

This work has confirmed the occurrence of four species in KSA previously reported as occurring in “Arabia”: *Pseudoathyreus flavohirtus* (Bolboceratidae) (Paulian, 1980); *Saprinus figuratus* (Histeridae) (Penati & Vienna, 2006); *Sphenoptera gahani* (Buprestidae) (Kerremans 1913); and *Syntomus lateralis* (Carabidae) (Schatzmayr 1936). The holotypes of three species originated from RKNP: *Boromorphus saudicus* Schawaller, Al Dhafer & Fadl, 2013; *Dicronychus latifahae* Al Dhafer & Platia, 2013 and *Reichardtiolus aldhaferi* Lackner, 2014.

In particular, *Adelostoma subtile arabicum* and *Adelostoma subtile sabulosum*, described from KSA by Kaszab (1981), but the characters for the differentiation of the two subspecies and the nominate *Adelostoma subtile subtile* Reitter that proposed by Kaszab (1981) are not useful for separation, and accordingly, this taxon is treated here as *Adelostoma subtile*.

It is expected that the results of this study of the beetle fauna of RKNP will be used for future studies related to long-term monitoring of the beetle fauna for evaluating impacts of increased use by the growing population of nearby Riyadh and impact of climate change (Bale et al. 2002).

### List of species

#### Suborder: Adephaga

##### Carabidae

###### Subfamily: Cicindelinae

####### Myriochila melancholica

Taxon classificationAnimaliaColeopteraCarabidae

(Fabricius, 1798)

######## World distribution.

**Africa**: AO, BF, BJ, BW, CD, CF, CG, CI, CM, CV, ER, ET, GH, GM, GN, GQ, GW, KE, MG, MS, MW, MZ, NA, NG, SD, SL, SN, SO, TD, TO, TZ, ZA, ZM, ZW. **Asia**: AE, AF, AZ, BH, CN, CY, EG (Sinai), IL, IN, IQ, IR, JO, KG, KW, KZ, LB, NP, OM, PK, QA, SA, SY, TJ, TM, TR, UZ, YE. **Europe**: AL, AM, ES, FR, GE, GR, IT, MT, NO (Svalbard), PT. **North Africa**: DZ, EG, ES (Canary Island), LY, MA, TN.

######## General distribution.

AFR_MAD_ORR_PAL_SAR_SJP.

######## Local distribution.

AS, NJ (Britton 1948; Cassola and Schneider 1997)

######## Collecting month and method.

A very rare species. The adults were collected by LT during V-VI.

###### Subfamily: Carabinae

####### Calosoma imbricatum

Taxon classificationAnimaliaColeopteraCarabidae

Klug, 1832

######## World distribution.

**Africa**: DJ, SD, TD (Britton 1948). **Asia**: AE, QA, SA, YE. **North Africa**: EG. Widespread all over Africa, the Arabian Peninsula and south Asia (Felix 2009).

######## General distribution.

AFR_ORR_SAR.

######## Local distribution.

AS, MK (Britton 1948), BA (El-Hawagry et al. 2013), EP, JZ, RI (Heinertz 1979).

######## Collecting month and method.

A very rare species collected only by LT through III-IV.

####### Calosoma olivieri

Taxon classificationAnimaliaColeopteraCarabidae

Dejean, 1831

######## World distribution.

**Asia**: AE (Felix 2009), AZ, IQ, IR, JO, PK, SA, SY, TM, UZ. **Europe**: MT, PT (Azores). **North Africa**: DZ, EG, ES (Canary Island), LY, MA, TN.

######## General distribution.

PAL_SAR.

######## Local distribution.

MK (Britton 1948), RI (Heinertz 1979).

######## Collecting month and method.

Very rare species. The beetles were collected by HP, and by PT under canopy of *Rhazya stricta* in II.

###### Subfamily: Scaritinae

####### Distichus planus

Taxon classificationAnimaliaColeopteraCarabidae

(Bonelli, 1813)

######## World distribution.

**Asia**: AZ, IQ, IR, JO, KZ, PK, SA, SY, TJ, TM, UZ, YE. **Europe**: ES, FR, GE, GR, IT, MT, PT. **North Africa**: EG, MA, TU.

######## General distribution.

PAL_SAR.

######## Local distribution.

RI (Balkenohl 1994).

######## Collecting month and method.

A frequent species that was collected HP, LT and PT during II, IV-VI, VIII, X, and XII.

####### Dyschirius beludscha ganglbaueri

Taxon classificationAnimaliaColeopteraCarabidae

Znojko, 1927

######## World distribution.

**Asia**: AE (Felix 2009), AF, EG (Sinai), IL, IQ, IR, KZ, MN, PK, SY, TJ, TM. **North Africa**: DZ, EG, MA, TU. New to KSA.

######## General distribution.

PAL_SAR.

######## Collecting month and method.

Very rare species that was collected by LT during IV.

####### Scarites procerus eurytus

Taxon classificationAnimaliaColeopteraCarabidae

Fischer von Waldheim, 1828

######## World distribution.

**Asia**: AF, IQ, IR, KG, KW, KZ, PK, SA, SY, TM, TR, UZ. **Europe**: ES, GE, GR, IT, PT, RU. **North Africa**: DZ, EG, LY, MA, TN.

######## General distribution.

PAL_SAR.

######## Local distribution.

EP, QS, RI (Balkenohl 1994), MK (Beccari 1971).

######## Collecting month and method.

Very rare species. The adults were collected by HP and PT during V.

###### Subfamily: Siagoninae

####### Siagona europaea

Taxon classificationAnimaliaColeopteraCarabidae

Dejean, 1826

######## World distribution.

**Asia**: AF, CY, EG (Sinai), IL, IN, IQ, IR, JO, KZ, LB, PK, SA, SY, TJ, TM, TR, UZ, YE. **Europe**: AL, AM, AZ, BG, ES, GE, GR, HR, IT, MK, PT, RU.

######## General distribution.

ORR_PAL_SAR.

######## Local distribution.

MK (Britton 1948), RI (Al Dhafer et al. 2016).

######## Collecting month and method.

A rare species. The beetles were collected by HP, LT and PT through IV-V, VII and XII.

###### Subfamily: Melaeninae

####### Cymbionotum pictulum

Taxon classificationAnimaliaColeopteraCarabidae

(Bates, 1874)

######## World distribution.

**Africa**: SD. **Asia**: Af, IQ, IR, KZ, SA, TM, TR. **E**: RU.

######## General distribution.

AFR_PAL_SAR.

######## Local distribution.

MK (Britton 1948; Ball and Shpeley 2005), RI (Al Dhafer et al. 2016).

######## Collecting month and method.

Frequent species that was collected by HP and LT during II-V and XII. HP.

####### Cymbionotum semelederi

Taxon classificationAnimaliaColeopteraCarabidae

(Chaudoir, 1864)

######## World distribution.

**Africa**: MR, NE, SD, SO, TD. **Asia**: AE (Felix 2009), AF, AZ, CY, IQ, IR, JO, KW, KZ, PK, SA, SY, TM, TR, UZ, YE. **Europe**: AM, GE, RU. **North Africa**: DZ, EG, MA, TN.

######## General distribution.

AFR_PAL_SAR.

######## Local distribution.

EP, RI (Basilewsky 1979), MK (Britton 1948).

######## Collecting month and method.

Frequent species. It was collected by LT and PT during II-V and XII.

###### Subfamily: Trechinae

####### Bembidion wittmeri

Taxon classificationAnimaliaColeopteraCarabidae

(Basilewsky, 1979)

######## World distribution.

**Asia**: SA.

######## General distribution.

END.

######## Local distribution.

RI (Basilewsky 1979).

######## Collecting month and method.

Common species. Its specimens were collected by HP, LT and PT through I-V and XII.

####### Tachyura lucasi

Taxon classificationAnimaliaColeopteraCarabidae

(Jacquelin du Val, 1852)

######## World distribution.

**Africa**: CD, CF, CM, ET, GQ, NG, ZA. **Asia**: CY, IL, IR, LB, SY, TR. **Europe**: ES, IT, PT. **North Africa**: DZ, EG, MA, TN. New to Arabian Peninsula.

######## General distribution.

AFR_MAD_PAL_SAR.

######## Collecting month and method.

A rare species. The beetles were collected by LT through IV-V and VII.

###### Subfamily: Brachininae

####### Brachinus crepitans

Taxon classificationAnimaliaColeopteraCarabidae

(Linné, 1758)

######## World distribution.

**Asia**: AZ, CY, IQ, IR, KG, KZ, SY, TJ, TM, TR, UZ. **Europe**: AL, AM, AT, BA, BE, BG, BY, CH, CZ, DE, DK, EE, ES, FI, FR, GB, GE, GR, HR, HU, IE, IT, LT, LU, LV, MD, NL, NO, PL, PT, RO, RS, RU, SE, SI, SK, UA. New to Arabian Peninsula.

######## General distribution.

PAL_SAR.

######## Collecting month and method.

Rare species. It was collected by HP during IV and XII; and by LT during V.

####### Brachinus nobilis

Taxon classificationAnimaliaColeopteraCarabidae

Dejean, 1831

######## World distribution.

**Asia**: IL, IQ, IR, SA, SY, TR, YE. **North Africa**: DZ, MA, TN.

######## General distribution.

SAR.

######## Local distribution.

MK (Britton 1948), RI (Basilewsky 1979).

######## Collecting month and method.

Rare species. The adults were collected by HP under debris and stones around temporary fresh water pool during XII.

###### Subfamily: Harpalinae

####### Amara aulica

Taxon classificationAnimaliaColeopteraCarabidae

(Panzer, 1796)

######## World distribution.

**Asia**: AZ, CN, KG, KZ, MN, RU, TJ, TM, TR, UZ. **Europe**: AL, AM, AT, BA, BE, BG, BY, CH, CZ, DE, DK, EE, ES, FI, FO, FR, GB, GE, GR, HR, HU, IE, IT, LI, LT, LV, MD, MK, NL, NO, PL, RO, RS, RU, SK, SE, SI, UA. **North Africa**: ES (Canary Island). New to Arabian Peninsula.

######## General distribution.

PAL_SAR_SJP.

######## Collecting month and method.

A frequent species that was collected HP and PT through I-IV and XII.

####### Amara maindroni

Taxon classificationAnimaliaColeopteraCarabidae

Bedel, 1907

######## World distribution.

**Asia**: AE (Felix 2009), AF, IQ, IR, JO, PK, SA, SY. **North Africa**: DZ, MA, TN.

######## General distribution.

SAR.

######## Local distribution.

RI (Hieke 1993).

######## Collecting month and method.

Very rare species. The adult was very rare and collected by Lt in XI.

####### Anthia duodecimguttata

Taxon classificationAnimaliaColeopteraCarabidae

Bonelli, 1813

######## World distribution.

**Asia**: AE (Felix 2009), IQ, IR, JO, KW, OM, QA, SA, YE. **North Africa**: EG.

######## General distribution.

SAR.

######## Local distribution.

AS, JZ, MK, RI (Britton 1948; Beccari 1971; Heinertz 1979).

######## Collecting month and method.

Very rare species. The adult beetles were collected by HP and LT at sandy area during IV.

####### Calodromius mayeti

Taxon classificationAnimaliaColeopteraCarabidae

(Bedel, 1907)

######## World distribution.

**Asia**: AE (Felix 2009), IR, SA. **North Africa**: MA, TN.

######## General distribution.

SAR.

######## Local distribution.

MD (Mateu 1986).

######## Collecting month and method.

Very rare species that was collected by HP under stones in IV.

####### Chlaenius flavipes

Taxon classificationAnimaliaColeopteraCarabidae

Menetries, 1832

######## World distribution.

**Asia**: AF, AZ, IQ, IR, KG, KZ, TJ, TM, TR, UZ. **Europe**: AL, AM, BA, BG, GE, GR, HR, HU, MD, MK, RO, RS, RU, UA. New to Arabian Peninsula.

######## General distribution.

PAL_SAR.

######## Collecting month and method.

Very rare species that was collected by HP under plant debris during XII.

####### Harpalus affinis

Taxon classificationAnimaliaColeopteraCarabidae

(Schrank, 1781)

######## World distribution.

**Asia**: AZ, CN, CY, IL, IR, KG, KP, KZ, MN, TR. **Australia**: AU, NZ. **Europe**: AD, AL, AM, AT, BA, BE, BG, BY, CH, CZ, DE, DK, EE, ES, FI, FR, GB, GE, GR, HR, HU, IE, IT, LI, LT, LU, LV, MD, MK, NL, NO, PL, PT, RO, RS, RU, SE, SI, SK, UA. **North America**: CA, US. New to Arabian Peninsula.

######## General distribution.

SCO.

######## Collecting month and method.

A rare species. The specimens were collected by HP under stones and plant debris during II, IV and XII.

####### Merizomena buettikeri

Taxon classificationAnimaliaColeopteraCarabidae

(Mateu, 1986)

######## World distribution.

**Asia**: SA.

######## General distribution.

END.

######## Local distribution.

MD (Mateu 1986).

######## Collecting month and method.

Very rare species. It was collected by LT in IV and VI.

####### Microlestes discoidalis

Taxon classificationAnimaliaColeopteraCarabidae

(Fairmaire, 1892)

######## World distribution.

**Africa**: ER, KE, MR, NE, SD, SO, TD. **Asia**: AE, AF, IL, IN, IR, SA, TR, YE.

######## General distribution.

AFR_ORR_SAR.

######## Local distribution.

MK (Britton 1948; Mateu 1979).

######## Collecting month and method.

Very rare species that was collected by LT through V.

####### Orthotrichus cymindoides

Taxon classificationAnimaliaColeopteraCarabidae

(Dejean, 1831)

######## World distribution.

**Asia**: AF, CN, IN, SY. **North Africa**: EG. New to Arabian Peninsula.

######## General distribution.

ORR_PAL_SAR_SJP.

######## Collecting month and method.

Frequent species. The adults were collected by HP, LT and PT during I-V, IX and XII.

####### Platytarus faminii faminii

Taxon classificationAnimaliaColeopteraCarabidae

(Dejean, 1826)

######## World distribution.

**Africa**: CV. **Asia**: AE (Felix 2009), AZ, CY, IL, IQ, KG, KZ, SA, SY, TM, TR, UZ, YE (Socotra). **Europe**: AM, ES, FR GR, IT, PT. **North Africa**: DZ, EG, ES (Canary Islands), LY, MA, TN.

######## General distribution.

AFR_PAL_SAR.

######## Local distribution.

AS, EP, RI (Mateu 1986).

######## Collecting month and method.

Very rare species that was collected by LT in III.

####### Poecilus wollastoni

Taxon classificationAnimaliaColeopteraCarabidae

(Wollaston, 1854)

######## World distribution.

**Asia**: IQ, KW, SA, YE. **North Africa**: DZ, EG, ES (Canary Islands), LY, MA, PT (Madeira Archipelago), TN.

######## General distribution.

SAR.

######## Local distribution.

It was recorded from Arabia without exact locality by Emden (1954) and a recent occurrence in KSA has been confirmed by Al Dhafer et al. (2016).

######## Collecting month and method.

Frequent species, which was collected by HP, LT and PT during I-II, IV-VI and XI-XII.

####### Stenolophus marginatus

Taxon classificationAnimaliaColeopteraCarabidae

Dejean, 1829

######## World distribution.

**Asia**: AE (Felix 2009), AF, AZ, CY, IL, IQ, IR, JO, KW, KZ, SA, SY, TJ, TM, TR, UZ. **Europe**: AL, AM, BA, BG, ES, FR, GE, GR, HR, IT, MK, PT, RS, UA. **North Africa**: DZ, EG, ES (Canary Islands), MA, PT (Madeira Archipelago), TN.

######## General distribution.

PAL_SAR.

######## Local distribution.

EP, RI (Basilewsky 1979).

######## Collecting month and method.

Frequent species. The beetles were collected by LT in IV-V, VII and IX.

####### Stenolophus pseudoobockianus

Taxon classificationAnimaliaColeopteraCarabidae

Felix & Muilwijk, 2009

######## World distribution.

**Asia**: AE (Felix 2009). New to KSA.

######## General distribution.

SAR.

######## Collecting month and method.

Very rare species that was collected by LT during IV.

####### Syntomus lateralis

Taxon classificationAnimaliaColeopteraCarabidae

(Motschulsky, 1855)

######## World distribution.

**Asia**: AE (Felix 2009), IL, IQ, IR, SA, SY. **North Africa**: DZ, EG, ES (Canary Islands), LY, MA, TN.

######## Local distribution.

Schatzmar (1936) mentioned Arabia among the distribution of this species in his work on Carabidae of Egypt, without given any further detailed about the locality.

######## General distribution.

SAR.

######## Collecting month and method.

Common species that was collected by HP, LT and PT in I-XII.

##### Dytiscidae

###### Subfamily: Dytiscinae

####### Eretes sticticus

Taxon classificationAnimaliaColeopteraDytiscidae

(Linné, 1767)

######## World distribution.

**Africa**: BW, KE, MG, NA, SD, ZA, ZW. **Asia**: AE, AZ, BT, CN, CY, EG (Sinai), IN, IQ, IR, JA, á, KW, NP, OM, PK, SA, SY, TM, TR, TW, YE. **Europe**: AM, BA, BG, ES, FR, GE, GR, HR, HU, IT, PT, RU, SI, UA, RS. **North Africa**: DZ, EG, ES (Canary Islands), LY, MA, PT (Madeira Archipelago), TN. **North America**: MX, PR, US. **South America**: EC, PE, VE.

######## General distribution.

COS.

######## Local distribution.

AS, EP, MD, RI (Brancucci 1979 1984).

######## Collecting month and method.

A frequent species. The adult beetles were collected by LT in IV-VI.

###### Subfamily: Hydroporinae

####### Hydroglyphus signatellus

Taxon classificationAnimaliaColeopteraDytiscidae

(Klug, 1834)

######## World distribution.

**Africa**: ET, KE, SD, SN. **Asia**: AE (Hájek and Brancucci 2011), AZ, CY, EG (Sinai), IL, IQ, IR, JO, KW, KZ, PK, SA, SY, TJ, TM, TR, UZ, YE. **Europe**: AM, BA, ES, GE, GR, HR, IT, RS, RU. **North Africa**: DZ, EG, LY, MA, TN.

######## General distribution.

AFR_PAL_SAR.

######## Local distribution.

EP, RI (Brancucci 1979 1984).

######## Collecting month and method.

Very rare species that was collected by LT during V.

####### Hygrotus inscriptus

Taxon classificationAnimaliaColeopteraDytiscidae

(Sharp, 1882)

######## World distribution.

**Asia**: AE, IQ, IR, KW, SA, SY, TM, UZ. **North Africa**: EG. The distribution is updated from Hájek and Brancucci (2011).

######## General distribution.

SAR.

######## Local distribution.

EP (Brancucci 1984).

######## Collecting month and method.

Frequent species that was collected by LT in VI.

#### Suborder: Polyphaga

##### Hydrophilidae

###### Subfamily: Hydrophilinae

####### Hydrochara flavipalpis

Taxon classificationAnimaliaColeopteraHydrophilidae

(Boheman, 1851)

######## World distribution.

**Asia**: OM, YE. New to KSA.

######## General distribution.

SAR.

######## Collecting month and method.

A rare species, which was collected by HP during V and XII.

##### Histeridae

###### Subfamily: Abraeinae

####### Teretrius pulex

Taxon classificationAnimaliaColeopteraHisteridae

Fairmaire, 1877

######## World distribution.

**Africa**: MR, NE, SN. **Asia**: AE (Kanaar 2007), OM, SA, SY. **North Africa**: DZ, EG, TN.

######## General distribution.

AFR_SAR.

######## Local distribution.

EP (Mazur 1994; Penati and Vienna 2006).

######## Collecting month and method.

Very rare species that was collected by BV on branches of *Ziziphus nummularia* during V.

###### Subfamily: Sapriniae

####### Pholioxenus sp.1

Taxon classificationAnimaliaColeopteraHisteridae

######## Collecting month and method.

A rare species. The specimens were collected by PT under canopy of *Acacia ehrenbergiana*, *Acacia gerrardii* and *Rhazya stricta* during IV-V and X.

####### Pholioxenus sp.2

Taxon classificationAnimaliaColeopteraHisteridae

######## Collecting month and method.

Frequent species. The beetles were collected by PT under canopies of *Acacia ehrenbergiana*, *Acacia gerrardii*, *Calotropis procera*, *Lycium shawii*, *Rhazya stricta* and *Ziziphus nummularia*; and by LT through V- XI.

####### Reichardtiolus aldhaferi

Taxon classificationAnimaliaColeopteraHisteridae

Lackner, 2014

######## World distribution.

**Asia**: SA (Lackner 2014).

######## General distribution.

END.

######## Local distribution.

RI (Lackner 2014).

######## Collecting month and method.

A rare species. The beetles were collected by HP and LT during I-II and XII.

####### Saprinus chalcites

Taxon classificationAnimaliaColeopteraHisteridae

(Illiger, 1807)

######## World distribution.

**Africa**: AO, BF, ET, GM, KE, ML, MR, NA, SD, SN, SO, TZ. **Asia**: AE, AF, CY, ID, IL, IN, IQ, IR, JO, KW, KZ, MM, MN, OM, PK, SA, TR, YE. **Australia**: AU. **Europe**: ES, FR, GR, IT, MT, PT, RU. **North Africa**: DZ, EG, ES (Canary Islands), LY, MA, PT (Madeira Archipelago), TN. **South America**: AR.

######## General distribution.

SCO (Penati and Vienna 2006).

######## Local distribution.

AS, BA, JZ, MK, NJ, RI (Kryzhanovskij 1979; Mazur 1994; Penati and Vienna 2006).

######## Collecting month and method.

Very rare species. The adults were collected by PT under canopies of *Acacia ehrenbergiana*, *Lycium shawii* and *Ziziphus nummularia* during IV and VII.

####### Saprinus confalonierii

Taxon classificationAnimaliaColeopteraHisteridae

G. Müller, 1933

######## World distribution.

**Africa**: MR. **Asia**: AE (Kanaar 2007), OM, SA. **North Africa**: DZ, EG, LY, TN.

######## General distribution.

AFR_SAR.

######## Local distribution.

AS, EP, HA, NB, NJ, RI (Mazur 1994; Penati and Vienna 2006).

######## Collecting month and method.

Very rare species that was collected by LT in X.

####### Saprinus figuratus

Taxon classificationAnimaliaColeopteraHisteridae

Marseul, 1855

######## World distribution.

**Asia**: IL, JO, SA, SY. **North Africa**: DZ, EG, ES (Canary Islands), LY, MA, TN.

######## General distribution.

SAR.

######## Local distribution.

Just recorded from Arabia (Penati and Vienna 2006).

######## Collecting month and method.

Very rare species that was collected by HP during II.

####### Saprinus moyses

Taxon classificationAnimaliaColeopteraHisteridae

Marseul, 1862

######## World distribution.

**Asia**: IR, KW, SA, SY, TR. **Europe**: GR. **North Africa**: DZ, EG, ES (Canary Islands), LY, MA, TN.

######## General distribution.

PAL_SAR.

######## Local distribution.

HA (Mazur 1994; Penati and Vienna 2006).

######## Collecting month and method.

Very rare species. The beetles were collected by BT and LT during V, VII and X.

####### Saprinus muelleri

Taxon classificationAnimaliaColeopteraHisteridae

Mazur, 1997

######## World distribution.

**Asia**: AE (Kanaar 2007), AF, PK, SA.

######## General distribution.

SAR.

######## Local distribution.

EP, MK, RI (Penati and Vienna 2006).

######## Collecting month and method.

Very rare species that was collected by LT during I-II.

####### Saprinus sp.

Taxon classificationAnimaliaColeopteraHisteridae

######## Collecting month and method.

A rare species. The adults were collected by LT during V-VI and X.

####### Xenonychus tridens

Taxon classificationAnimaliaColeopteraHisteridae

(Jacquelin Duval, 1853)

######## World distribution.

**Africa**: CV, MR, NG, TD. **Asia**: AE (Kanaar 2007), CY, IL, OM, SA, SY, TR.

**Europe**: ES, FR, GR, IT, PT. **North Africa**: DZ, EG, ES (Canary Islands), LY, MA, TN.

######## General distribution.

AFR_PAL_SAR.

######## Local distribution.

EP, MK (Mazur 1994; Penati and Vienna 2006), RI (Al Dhafer et al. 2016).

######## Collecting month and method.

Very rare species that was recorded by PT under canopy of *Acacia ehrenbergiana* during IV.

####### Xenophilothis choumovitchi

Taxon classificationAnimaliaColeopteraHisteridae

(Thérond & Hollande, 1965)

######## World distribution.

**Asia**: AE (Kanaar 2007), OM, SA. **North Africa**: DZ.

######## General distribution.

SAR.

######## Local distribution.

EP, RI (Penati and Vienna 2006).

######## Collecting month and method.

Very rare species, which was collected by LT in V.

####### Zorius sp.

Taxon classificationAnimaliaColeopteraHisteridae

######## Collecting month and method.

A rare species. The adults were collected by PT under canopies of *Acacia gerrardii*, *Calotropis procera*, *Lycium shawii* and *Rhazya stricta* during III-IV.

###### Subfamily: Histerinae

####### Atholus bimaculatus

Taxon classificationAnimaliaColeopteraHisteridae

(Linnaeus, 1758)

######## World distribution.

**Africa**: BF, CF, CM, DJ, KE, MR, SN, TD. **Asia**: AE (Kanaar 2007), AF, CN, CY, IL, IN, IQ, IR, JO, JP, KG, KP, KR, KZ, MM, OM, RU, SA, SY, TJ, TM, UZ, YE. **Europe**: AL, AM, AT, BA, BE, BG, BY, CH, CZ, DE, DK, EE, ES, FI, FR, GB, GE, GR, HR, HU, IE, IT, LI, LV, NL, NO, PL, PT, RO, RS, RU, SE, SI, SK, UA. **North Africa**: DZ, ES (Canary Islands), LY, MA, TN. **North America**: CA, US. **South America**: AR, BR, CL.

######## General distribution.

SCO (Penati and Vienna 2006).

######## Local distribution.

AS (Kryzhanovskij 1979), RI (Al Dhafer et al. 2016).

######## Collecting month and method.

Very rare species that was collected by PT under canopy of *Acacia ehrenbergiana* during IV.

##### Leiodidae

###### Subfamily: Leiodinae

####### Chobautiella anisotomoides

Taxon classificationAnimaliaColeopteraLeiodidae

(Fairmaire, 1876)

######## World distribution.

**Asia**: AE (Švec 2010a). **North Africa**: DZ, ES (Canary Islands), MA, TN (Švec 2010). New to KSA.

######## General distribution.

SAR.

######## Collecting month and method.

Very rare species. The specimens were collected by LT in XII.

##### Staphylinidae

###### Subfamily: Pselaphinae

####### Ctenisomorphus major

Taxon classificationAnimaliaColeopteraLeiodidae

(Raffray, 1877)

######## World distribution.

**Africa**: ET. **Asia**: AE (Besuchet and Cuccodoro 2011), EG (Sinai), IL, IR, JO, SA, TR, YE. **North Africa**: DZ, EG, TN.

######## General distribution.

AFR_SAR.

######## Local distribution.

AS, BA, EP, MD, RI (Besuchet 1981).

######## Collecting month and method.

Very rare species that was collected by LT in IV-V and VII.

####### Enoptostomus arabicus

Taxon classificationAnimaliaColeopteraLeiodidae

Besuchet & Cuccodoro, 2011

######## World distribution.

**Asia**: AE (Besuchet and Cuccodoro 2011). New to KSA.

######## General distribution.

SAR.

######## Collecting month and method.

Very rare species, which was collected by LT during VII.

###### Subfamily: Tachyporinae

####### Sepedophilus sp.

Taxon classificationAnimaliaColeopteraLeiodidae

######## Collecting month and method.

Very rare species. It was collected by PT under canopy of *Ziziphus nummularia* in III.

###### Subfamily: Aleocharinae

####### Atheta atramentaria

Taxon classificationAnimaliaColeopteraLeiodidae

(Gyllenhal, 1810)

######## World distribution.

**Africa**: MR, ZA. **Asia**: AZ, CN, CY, IN, IR, JP, KP, KZ, NP, PK, RU, TR. **Europe**: AL, AT, BE, BG, CH, CZ, DE, DK, EE, ES, FI, FO, FR, GB, GE, GR, HU, IE, IS, IT, LT, LU, NL, NO, PL, PT, RU, SE, SK. **North Africa**: DZ, EG, ES (Canary Islands), MA, PT (Madeira Archipelago), TN. New to Arabian Peninsula.

######## General distribution.

AFR_PAL_ORR_SAR_SJP.

######## Collecting month and method.

Rare species. The specimens of this species were collected by PT under canopies of *Acacia ehrenbergiana*, *Acacia gerrardii*, *Lycium shawii*, *Rhazya stricta*, and *Ziziphus nummularia* in I-II and X-XI; and by SW on branches *of Calotropis procera* through VII.

###### Subfamily: Oxytelinae

####### Bledius niloticus

Taxon classificationAnimaliaColeopteraLeiodidae

Erichson, 1840

######## World distribution.

**Africa**: AO, ET, SN, ZA. **Asia**: CN, IL, IN, JP, KP, KR, LB, LK, SA (Beccari 1971), SY. **North Africa**: DZ, EG, MA, TN.

######## General distribution.

AFR_ORR_PAL_SAR_SJP.

######## Local distribution.

RI (Beccari 1971).

######## Collecting month and method.

Very rare species that was collected by LT during X.

####### Carpelimus pusillus

Taxon classificationAnimaliaColeopteraLeiodidae

(Gravenhorst, 1802)

######## World distribution.

**Asia**: CY, IR, KZ, RU, TR. **Australia**: AU. **Europe**: AL, AM, AT, BA, BE, BG, CH, CZ, DE, DK, EE, ES, FI, FR, GB, GE, GR, HR, HU, IE, IS, IT, LT, LV, NL, NO, PL, PT, RO, RU, SE, SI, SK, UA. **North Africa**: DZ, EG, ES (Canary Islands), PT (Madeira Archipelago). **North America**: US. New to Arabian Peninsula.

######## General distribution.

SCO.

######## Collecting month and method.

Very rare species, which was collected by LT through XII.

####### Carpelimus sp.

Taxon classificationAnimaliaColeopteraLeiodidae

######## Collecting month and method.

Very rare species that was collected by LT in VII.

###### Subfamily: Paederinae

####### Philonthus sp.

Taxon classificationAnimaliaColeopteraLeiodidae

######## Collecting month and method.

Very rare species that was collected by LT through VI.

##### Glaresidae

###### Glaresis arabica

Taxon classificationAnimaliaColeopteraGlaresidae

(Paulian, 1980)

####### World distribution.

**Asia**: OM, SA.

####### General distribution.

SAR.

####### Local distribution.

EP, RI (Paulian 1980).

####### Collecting month and method.

Very rare species that was collected by LT during V.

###### Glaresis sp.

Taxon classificationAnimaliaColeopteraGlaresidae

####### Collecting month and method.

Very rare species that was collected by LT in IV.

##### Bolboceratidae

###### Subfamily: Bolboceratinae

####### Pseudoathyreus flavohirtus

Taxon classificationAnimaliaColeopteraBolboceratidae

(Wlaker, 1871)

######## World distribution.

**Asia**: Arabia

######## General distribution.

SAR.

######## Local distribution.

Paulian (1980) recorded this species from Arabia without exact locality.

######## Collecting month and method.

Very rare species that was collected by LT in IV.

##### Hybosoridae

###### Subfamily: Hybosorinae

####### Hybosorus illigeri

Taxon classificationAnimaliaColeopteraHybosoridae

Reiche, 1853

######## World distribution.

**Africa**: KM, MG, UG. **Asia**: AF, AZ, CN, CY, EG (Sinai), IL, IN, IQ, IR, JO, PK, SA, SY, TJ, TM, TR, UZ, YE, VN. **Europe**: AM, BG, ES, FR, GE, GR, HR, IT, MK, PT, RO, RS, RU, UA. **North Africa**: DZ, EG, LY, MA, TN. **North America**: CU, HT, MX, NI, US. **South America**: VE.

######## General distribution.

SCO.

######## Local distribution.

EP, MD, MK, NJ, RI (Beccari 1971, Kuijten 1980).

######## Collecting month and method.

An abundant species. The adult beetles were collected by HP, LT and PT through II, IV-VIII.

##### Scarabaeidae

###### Subfamily Eremazinae

####### Eremazus giganteus

Taxon classificationAnimaliaColeopteraScarabaeidae

Král, 2015

######## World distribution.

**Asia**: AE (Král and Batelka 2015). New to KSA.

######## General distribution.

SAR.

######## Collecting month and method.

Very rare species that collected by LT during V.

####### Eremazus unistriatus

Taxon classificationAnimaliaColeopteraScarabaeidae

Mulsant, 1851

######## World distribution.

**Africa**: NE, SD. **Asia**: AE, AF, IL, IN, IQ, IR, KZ, PK, SA, TJ, TM, TR, UZ. **Europe**: AM, GE. **North Africa**: DZ, EG, ES (Canary Islands), LY, MA, TN.

######## General distribution.

AFR_ORR_PAL_SAR.

######## Local distribution.

EP, HA, MD, MK, QS, RI (Pittino 1984).

######## Collecting month and method.

A frequent species. The adults of this species were collected by PT under canopy of *Acacia ehrenbergiana*, and by HP and LT during I-II, IV-VII and IX.

###### Subfamily: Aphodiinae

####### Aphodius adustus

Taxon classificationAnimaliaColeopteraScarabaeidae

Klug, 1855

######## World distribution.

**Africa**: CD, CM, DJ (Pittino 1984), GW, KE, MZ, NA, SD (Balthasar 1972), SN, TD, TZ. **Asia**: SA, YE.

######## General distribution.

AFR_SAR.

######## Local distribution.

The species was reported from Arabia without exact locality (Pittino 1984) and recently its occurrence in KSA: RI has been confirmed by Abdel-Dayem et al. (2016).

######## Collecting month and method.

Very rare species that was collected by LT during IV.

####### Aphodius arabicus

Taxon classificationAnimaliaColeopteraScarabaeidae

Harold, 1875

######## World distribution.

**Asia**: EG (Sinai), IL, IR, KW, SA, YE. **North Africa**: EG

######## General distribution.

SAR.

######## Local distribution.

EP, JZ, MD, MK, RI (Pittino 1984).

######## Collecting month and method.

Common species that was collected by LT through V-VII and IX-X.

####### Aphodius beluchistanicus

Taxon classificationAnimaliaColeopteraScarabaeidae

Petrovitz, 1962

######## World distribution.

**Asia**: IQ, IR, SA.

######## General distribution.

SAR.

######## Local distribution.

EP, RI (Pittino 1984).

######## Collecting month and method.

A rare species, which was collected by LT in I and XII.

####### Aphodius chobauti

Taxon classificationAnimaliaColeopteraScarabaeidae

Clouët, 1896

######## World distribution.

**Asia**: IL, IQ, IR, JO, SA. **North Africa**: DZ, EG, LY, MA, TN.

######## General distribution.

SAR.

######## Local distribution.

MD, QS, RI (Pittino 1984).

######## Collecting month and method.

Rare species. The specimens were collected by BV on branches of *Acacia gerrardii*, and by HP and LT during IV-V and XI.

####### Aphodius ictericus ghardimaouensis

Taxon classificationAnimaliaColeopteraScarabaeidae

Balthasar, 1929

######## World distribution.

**Asia**: CY, IL, IR, JO, LB, SA, SY, TR. **Europe**: ES, FR, GR, HR, IT, MT, PT. **North Africa**: DZ, EG, ES (Canary Islands), LY, MA, TN.

######## General distribution.

PAL_SAR.

######## Local distribution.

EP (Pittino 1984).

######## Collecting month and method.

Abundant species that was collected by LT during I, III and IX.

####### Aphodius lividus

Taxon classificationAnimaliaColeopteraScarabaeidae

(Olivier, 1789)

######## World distribution.

**Africa**: MG, NA. **Asia**: CN, CY, EG (Sinai), IL, IR, KG, KW, KZ, LB, MN, NP, OM, SA, SY, TJ, TM, TR, TW, UZ. **Australia**: AU, NZ, PG. **Europe**: AL, AM, AT, BA, BE, BY, CH, CZ, DE, EE, ES, FI, FR, GB, GE, GR, HR, HU, IT, LT, LV, MK, MT, NL, PL, PT, RO, RS, RU, SE, SI, SK, UA. **North Africa**: DZ, EG, ES (Canary Islands), LY, MA, PT (Madeira Archipelago), TN. **North America**: CU, GT, MX, NI, PA, US.

######## General distribution.

SOC.

######## Local distribution.

AS, BA, EP, JZ, MD, MK, QS, RI (Beccari 1971, Pittino 1984; El-Hawagry et al. 2013).

######## Collecting month and method.

Common species. The beetles were collected by LT through IV-VII and IX-XII.

####### Aphodius luridus

Taxon classificationAnimaliaColeopteraScarabaeidae

(Fabricus, 1775)

######## World distribution.

**Asia**: CN, CY, IL, IR, KG, KZ, RU, SA, SY, TJ, TM, TR. **Europe**: AL, AM, AT, BA, BE, BG, BY, CH, DE, DK, EE, ES, FI, FR, GB, GE, GR, HR, HU, IE, IT, LT, LV, MK, NL, NO, PL, PT, RO, RS, RU, SE, SI, SK, TR, UA. **North Africa**: MA, TN.

######## General distribution.

PAL_SAR_SJP.

######## Local distribution.

RI (Abdel-Dayem et al. 2016).

######## Collecting month and method.

Very rare species that was collected by LT in I and X.

####### Aphodius pruinosus

Taxon classificationAnimaliaColeopteraScarabaeidae

Reitter, 1892

######## World distribution.

**Africa**: MR. **Asia**: AF, IL, IQ, IR, KG, KW, KZ, LB, OM, PK, SA, TJ, TM, TR, UZ. **Europe**: RU. **North Africa**: DZ, EG, LY, MA, TN.

######## General distribution.

AFR_PAL_SAR.

######## Local distribution.

BA, EP, HA, JZ, MD, MK, QS, RI (Pittino 1984).

######## Collecting month and method.

An abundant species. The adults were collected by PT by PT under canopies of *Lycium shawii* and *Rhazya stricta*; and by LT through II-VI and IX-X.

####### Aphodius rendallii

Taxon classificationAnimaliaColeopteraScarabaeidae

(Wollaston, 1867)

######## World distribution.

**Asia**: IL, IQ, JO, SA. **North Africa**: DZ, EG, LY, MA, TN.

######## General distribution.

SAR.

######## Local distribution.

EP, HA, QS, RI (Pittino 1984) [under the name *Aphodius opacior* D. Koshantschikov 1894] (Král and Batelka 2015).

######## Collecting month and method.

Common species. The specimens of this species were collected by PT under canopies of *Acacia ehrenbergiana*, *Acacia gerrardii*, *Lycium shawii*, *Rhazya stricta* and *Ziziphus nummularia*; and by LT through IV-V and IX-X.

####### Aphodius translucidus

Taxon classificationAnimaliaColeopteraScarabaeidae

Petrovitz, 1961

######## World distribution.

**Asia**: AF, IN, IQ, IR, OM, PK, RU, SA, TM. **North Africa**: EG (Pittino 1984).

######## General distribution.

ORR_SAR.

######## Local distribution.

EP, MD, MK, RI (Pittino 1984).

######## Collecting month and method.

Common species that was collected by LT in IV-V and IX-XI.

####### Aphodius wollastoni iranicus

Taxon classificationAnimaliaColeopteraScarabaeidae

Balthasar, 1946

######## World distribution.

**Asia**: EG (Sinai), IN, IQ, IR, JO, OM, PK, SA, SY, YE.

######## General distribution.

ORR_SAR.

######## Local distribution.

EP, HA, MD, QS, RI (Pittino 1984).

######## Collecting month and method.

Abundant species. It was collected by PT under canopies of *Acacia ehrenbergiana* and *Calotropis procera*; and by HP and LT through I-V and XI-XII.

####### Granulopsammodius plicatulus

Taxon classificationAnimaliaColeopteraScarabaeidae

(Fairmeire, 1892)

######## World distribution.

**Africa**: ET, SD, SO. **Asia**: SA, YE. **North Africa**: DZ, EG, LY, MA.

######## General distribution.

AFR_SAR.

######## Local distribution.

AS, BA, EP, MD, MK, RI (Paulian 1980; Pittino 1984; El-Hawagry et al. 2013).

######## Collecting month and method.

Common species, which was collected by PT under canopy of *Ziziphus nummularia*; and by LT through IV-VIII.

####### Leiopsammodius laevicollis

Taxon classificationAnimaliaColeopteraScarabaeidae

(Klug, 1845)

######## World distribution.

**Africa**: CG, DJ, ER, ET, SD, SO. **Asia**: IL, SA, SY, YE. **North Africa**: DZ, EG, LY, MA, TN.

######## General distribution.

AFR_SAR.

######## Local distribution.

BA, HA, JZ, MD, MK, RI (Pittino 1984; El-Hawagry et al. 2013).

######## Collecting month and method.

A rare species that was collected by PT under canopy of *Lycium shawii* and *Rhazya stricta*; and by LT through IV-VI, IX and XII.

####### Pararhyssemus coluber

Taxon classificationAnimaliaColeopteraScarabaeidae

(Mayet, 1887)

######## World distribution.

**Africa**: SO. **Asia**: AF, EG (Sinai), IR, SA. **North Africa**: DZ, EG, LY, MA, TN.

######## General distribution.

AFR_SAR.

######## Local distribution.

AS, BA, HA, MD, MK, RI (Pittino 1984).

######## Collecting month and method.

Very rare species. The beetles were collected by LT in IV.

####### Pleurophorus arabicus

Taxon classificationAnimaliaColeopteraScarabaeidae

(Pittino & Mariani, 1986)

######## World distribution.

**Asia**: AZ, IR, JO, SA, TM. **Europe**: RU.

######## General distribution.

PAL_SAR.

######## Local distribution.

EP, QA, RI (Pittino 1984) [under the name *Pleurophorus anatolicus* Petrovitz 1961] (Král and Batelka 2015).

######## Collecting month and method.

A common species. The adult beetles were collected by PT under canopies of *Acacia ehrenbergiana*, *Acacia gerrardii*, *Calotropis procera* and *Rhazya stricta*; and by HP and LT through III-V and XII.

####### Pseudomothon sp.

Taxon classificationAnimaliaColeopteraScarabaeidae

######## Collecting month and method.

Rare species that was collected by LT during IV.

####### Rhyssemus brevitarsis

Taxon classificationAnimaliaColeopteraScarabaeidae

Pinttino, 1984

######## World distribution.

**Asia**: SA.

######## General distribution.

END.

######## Local distribution.

BA, JZ, MD, MK, QS, RI (Pittino 1984; El-Hawagry et al. 2013).

######## Collecting month and method.

Very rare species that was collected by LT during IV.

####### Rhyssemus granosus

Taxon classificationAnimaliaColeopteraScarabaeidae

(Klug & Erichson, 1842)

######## World distribution.

**Africa**: CM, CV, ET, KE, ML, MR, NE, NG, SD, SN, SO, TD, TZ. **Asia**: SA, YE. **North Africa**: EG.

######## General distribution.

AFR_SAR.

######## Local distribution.

AS, BA, EP, JZ, MD, MK, QS, RI (Beccari 1971, Pittino 1984; El-Hawagry et al. 2013).

######## Collecting month and method.

A rare species. The beetles were collected by LT through IV-V, VII and X.

####### Rhyssemus saoudi

Taxon classificationAnimaliaColeopteraScarabaeidae

Pittino, 1984

######## World distribution.

**Asia**: SA.

######## General distribution.

END.

######## Local distribution.

AS, BA, JZ, MK, RI (Pittino 1984; El-Hawagry et al. 2013; Abdel-Dayem et al. 2016).

######## Collecting month and method.

Common species. The adult beetles were collected by PT under canopies of *Calotropis procera*, *Lycium shawii*, *Rhazya stricta* and *Ziziphus nummularia*; and by HP and LT through III-V and VII-XI.

###### Subfamily Scarabaeinae

####### Metacatharsius inermis

Taxon classificationAnimaliaColeopteraScarabaeidae

(Laporte, 1840)

######## World distribution.

**Africa**: ER, ET, GM, KE, MR, SD, SN, SO, TD. **Asia**: IN, IQ, IR, PK, SA. **North Africa**: EG.

######## General distribution.

AFR_ORR_SAR.

######## Local distribution.

AS, EP, RI (Paulian 1980).

######## Collecting month and method.

Rare species, which was collected during V.

####### Scarabaeus bannuensis

Taxon classificationAnimaliaColeopteraScarabaeidae

A. Janssens, I940

######## World distribution.

**Africa**: MR, TD. **Asia**: IQ, IR, PK, SA. **North Africa**: DZ, LY, MA, TN.

######## General distribution.

AFR_SAR.

######## Local distribution.

RI (Ziani and Gudenzi 2012)

######## Collecting month and method.

Common species. The specimens were collected by PT under canopies of *Acacia ehrenbergiana*, *Acacia gerrardii*, *L shawii* and *Rhazya stricta*; and by HP, LT, SW during III-VIII.

####### Scarabaeus cristatus

Taxon classificationAnimaliaColeopteraScarabaeidae

Fabricius, 1775

######## World distribution.

**Africa**: ER, GN, MR, NE, SD, SN, TD. **Asia**: AE, AF, IL, IQ, IR, PK, SA. **North Africa**: EG, LY.

######## General distribution.

AFR_SAR.

######## Local distribution.

AS, EP, MK, RI (Paulian 1980).

######## Collecting month and method.

Very rare species. The specimens were collected by HP and LT during V and VIII.

###### Subfamily: Dynamopodinae

####### Orubesa plicifrons

Taxon classificationAnimaliaColeopteraScarabaeidae

(Fairmaire, 1897)

######## World distribution.

**Africa**: ET, NE, SN. **Asia**: SA (Paulian 1980). **North Africa**: MA.

######## General distribution.

AFR_SAR.

######## Local distribution.

RI (Paulian 1980).

######## Collecting month and method.

A common species. The adult beetles were collected by HP and LT in IV.

###### Subfamily Melolonthinae

####### Maladera insanabilis

Taxon classificationAnimaliaColeopteraScarabaeidae

(Brenske, 1894)

######## World distribution.

**Asia**: AE, AF, IL, IN, IQ, IR, JO, KW, NP, OM, PK, SA. **North Africa**: LY.

######## General distribution.

ORR_SAR.

######## Local distribution.

EP, RI (Ahrens 2000).

######## Collecting month and method.

Very rare species that was collected by LT during V, VII and XI.

####### Schizonycha buettikeri

Taxon classificationAnimaliaColeopteraScarabaeidae

Sabatinelli & Pontuale, 1998

######## World distribution.

**Asia**: OM, SA.

######## General distribution.

SAR.

######## Local distribution.

EP, HA, RI (Sabatinelli and Pontuale 1998).

######## Collecting month and method.

A rare species that was collected by LT during IV-V.

####### Schizonycha flavicornis

Taxon classificationAnimaliaColeopteraScarabaeidae

Brenske, 1898

######## World distribution.

**Africa**: SD, SO. **Asia**: SA, YE. **North Africa**: EG.

######## General distribution.

AFR_SAR.

######## Local distribution.

AS, BA, EP, JZ, MD, MK, RI (Sabatinelli and Pontuale 1998).

######## Collecting month and method.

Rare species, which was collected by LT in IV and X-XI.

####### Sphaerotrochalus somalicola

Taxon classificationAnimaliaColeopteraScarabaeidae

(Frey, 1960)

######## World distribution.

**Africa**: ET, SO. **Asia**: OM, SA, YE.

######## General distribution.

AFR_SAR.

######## Local distribution.

AS, BA, HA, JZ, MK, RI (Ahrens 2000).

######## Collecting month and method.

A common species. The specimens were collected by PT under canopies of *Acacia ehrenbergiana*, *Acacia gerrardii* and *Rhazya stricta*; and by LT during I, IV-V and X-XII.

###### Subfamily Rutelinae

####### Clipadoretus habibi

Taxon classificationAnimaliaColeopteraScarabaeidae

Král, 2015

######## World distribution.

**Asia**: AE (Král and Batelka 2015). New to KSA.

######## General distribution.

SAR.

######## Local distribution.

RI (Beccari 1971).

######## Collecting month and method.

A rare species that was recorded by LT in V-VII and IX.

####### Clipadoretus sp.

Taxon classificationAnimaliaColeopteraScarabaeidae

######## Collecting month and method.

Moderately common species that was collected by LT during V.

####### Phaeadoretus syriacus

Taxon classificationAnimaliaColeopteraScarabaeidae

(C. É. Blanchard, 1851)

######## World distribution.

**Asia**: IQ, IR. New to Arabian Peninsula.

######## General distribution.

SAR.

######## Collecting month and method.

Abundant species. The specimens were collected by PT under canopies of *Acacia ehrenbergiana*, *Acacia gerrardii*, *Lycium shawii* and *Ziziphus nummularia*; and by HP and LT through I-V.

###### Subfamily Dynastinae

####### Pentodon algerinus dispar

Taxon classificationAnimaliaColeopteraScarabaeidae

Baudi, 1870

######## World distribution.

**Africa**: ER. **Asia**: CY, IL, IQ, IR, JO, KW, OM, QA, SA, SY, YE. **Europe**: AM, GR.

######## General distribution.

AFR_PAL_SAR.

######## Local distribution.

EP, MK, RI (Endrödi 1980).

######## Collecting month and method.

Common species. The adults were collected by PT under canopies of *Rhazya stricta* and *Ziziphus nummularia*; and by HP and LT through IV-V and VIII-IX.

####### Podalgus cuniculus arabicus

Taxon classificationAnimaliaColeopteraScarabaeidae

Fairmaire, 1895

######## World distribution.

**Asia**: AE, EG (Sinai), IL, KW, OM, SA, YE. **North Africa**: EG.

######## General distribution.

SAR.

######## Local distribution.

EP, MK, RI (Endrödi 1980).

######## Collecting month and method.

A common species that was collected by HP and LT through IV-XI.

##### Dascillidae

###### Subfamily: Karumiinae

####### Karumia inaequalis

Taxon classificationAnimaliaColeopteraDascillidae

Pic, 1929

######## World distribution.

**Asia**: SA.

######## General distribution.

END.

######## Local distribution.

EP, RI (Wittmer 1979).

######## Collecting month and method.

Abundant species that was collected by PT under canopies of *Acacia ehrenbergiana*, and by LT during IV-IX.

##### Buprestidae

###### Subfamily: Julodinae

####### Julodis euphratica

Taxon classificationAnimaliaColeopteraBuprestidae

Laporte & Gory, 1835

######## World distribution.

**Asia**: AF, EG (Sinai), IQ, IR, JO, OM, SA.

######## General distribution.

SAR.

######## Local distribution.

EP, QS, RI (Bílý 1982 1985).

######## Collecting month and method.

It is a rare and was collected by BV, HP and VC during I and IV-VI.

###### Subfamily: Polycestinae

####### Acmaeoderella arabica

Taxon classificationAnimaliaColeopteraBuprestidae

Cobos, 1963

######## World distribution.

**Asia**: IL, IR, OM, SA.

######## General distribution.

SAR.

######## Local distribution.

RI (Cobos 1963).

######## Collecting month and method.

Very rare species, which was collected by BV on branches of *Ziziphus nummularia* during VI.

####### Xantheremia pantherina

Taxon classificationAnimaliaColeopteraBuprestidae

(Bílý, 1979)

######## World distribution.

**Asia**: IL, IQ, SA. **North Africa**: EG.

######## General distribution.

SAR.

######## Local distribution.

EP, RI (Bílý 1982 1985).

######## Collecting month and method.

A rare species. The specimens were collected by BV on branches of *Lycium shawii*, HP and by SW of *Rhazya stricta* during V, VII and VIII.

###### Subfamily: Chrysochroinae

####### Sphenoptera gahani

Taxon classificationAnimaliaColeopteraBuprestidae

Kerremans, 1913

######## World distribution.

**Asia**: Arabia (Kerremans 1913)

######## General distribution.

END.

######## Local distribution.

This species is described by Kerremans (1913) from Arabia, without any further information about the type locality.

######## Collecting month and method.

Very rare species that was collected only by BV on branches of *Acacia gerrardii* during X.

####### Sphenoptera magna

Taxon classificationAnimaliaColeopteraBuprestidae

Gory & Laporte, 1839

######## World distribution.

**Asia**: IL, IQ, IR, JO, SA, SY, TR.

######## General distribution.

PAL_SAR.

######## Local distribution.

EP, QS (Bílý 1980).

######## Collecting month and method.

Very rare species that was collected by HP during V.

###### Subfamily: Buprestinae

####### Anthaxia kneuckeri

Taxon classificationAnimaliaColeopteraBuprestidae

Obenberger, 1920

######## World distribution.

**Asia**: EG (Sinai), IL, JO, OM, SA.

######## General distribution.

SAR.

######## Local distribution.

AS, MK, RI (Bílý 1980), BA (El-Hawagry et al. 2013).

######## Collecting month and method.

Common species. The adults were collected by BV and VC on branches of *Acacia ehrenbergiana* and *Acacia gerrardii*, and by HP, LT and PT during IV-XI.

####### Anthaxia marginifera metallenscens

Taxon classificationAnimaliaColeopteraBuprestidae

Bílý, 1999

######## World distribution.

**Asia**: IL, SA.

######## General distribution.

SAR.

######## Local distribution.

RI (Bílý 1979).

######## Collecting month and method.

Very rare species that was collected by BV on branches of *Acacia ehrenbergiana* during IV.

###### Subfamily: Agrilinae

####### Agrilus desertus

Taxon classificationAnimaliaColeopteraBuprestidae

(Klug, 1829)

######## World distribution.

**Africa**: MR, SO, TD. **Asia**: IL, IR, JO, SA, YE. **North Africa**: DZ, EG, LY, MA, TN.

######## General distribution.

AFR_SAR.

######## Local distribution.

AS (Bílý 1982).

######## Collecting month and method.

A rare species. The specimens were collected by BV on branches of *Acacia ehrenbergiana*, *Acacia gerrardii* and *Lycium shawii*, and HP during IV-V, VIII and X.

####### Agrilus lituratus

Taxon classificationAnimaliaColeopteraBuprestidae

(Klug, 1829)

######## World distribution.

**Africa**: MR, SD, SN, TD (Bílý 1982). **Asia**: IL, IR, JO, SA, SY, YE. **North Africa**: DZ, EG, LY, MA, TN.

######## General distribution.

AFR_SAR.

######## Local distribution.

AS, BA, MK, RI (Bílý 1982).

######## Collecting month and method.

Frequent species that was collected by BV, SW and VC on branches of *Acacia ehrenbergiana*, *Acacia gerrardii* and *Calotropis procera*, and PT during IV-VII and IX.

####### Trachys latifrons

Taxon classificationAnimaliaColeopteraBuprestidae

Kerremans, 1907

######## World distribution.

**Africa**: ER, ET, SD. **Asia**: AE, SA, YE.

######## General distribution.

AFR_SAR.

######## Local distribution.

AS (Bílý 1979), RI (Al Dhafer et al. 2016).

######## Collecting month and method.

Very rare species. It was collected by PT under the canopy of *Ziziphus nummularia* during IV.

##### Heteroceridae

###### Subfamily: Heterocerinae

####### Augyles sericans

Taxon classificationAnimaliaColeopteraHeteroceridae

(Kiesenwetter, 1843)

######## World distribution.

**Asia**: IL, SY. **Europe**: AL, AT, BG, CH, CZ, DE, DK, ES, FR, GB, HR, HU, IT, NL, PL, RO, SE, SI, SK, UA. New to Arabian Peninsula.

######## General distribution.

PAL_SAR.

######## Collecting month and method.

Rare species that was collected by Lt during IV-V.

####### Augyles turanicus

Taxon classificationAnimaliaColeopteraHeteroceridae

(Reitter, 1887)

######## World distribution.

**Asia**: AE (Mascagni 2009), IL, IQ, IR, KZ, SY, TM, TR, UZ. **Europe**: GE. **North Africa**: DZ. New to KSA.

######## General distribution.

PAL_SAR.

######## Collecting month and method.

Very rare species, which was collected by LT in IV.

##### Elateridae

###### Subfamily: Agrypninae

####### Aeoloides grisescens

Taxon classificationAnimaliaColeopteraElateridae

(Germar, 1844)

######## World distribution.

**Africa**: SD, TD. **Asia**: AE, AF, AZ, CN, CY, IQ, IR, KG, KZ, MN, OM, PK, QA, SA, SY, TJ, TM, TR, UZ, YE. **Europe**: AM, GE, GR, RU, UA. **North Africa**: EG, LY, MA.

######## General distribution.

AFR_PAL_SAR_SJP.

######## Local distribution.

EP, JZ, QS, RI (Platia and Schimmel 1997).

######## Collecting month and method.

Common species. The adults were collected by PT under canopies of *Acacia ehrenbergiana*, *Lycium shawii*, *Rhazya stricta* and *Ziziphus nummularia*; and by HP and LT during I-VII and IX-X.

####### Aeoloides holzschuhi

Taxon classificationAnimaliaColeopteraElateridae

Platia & Schimmel, 1997

######## World distribution.

**Asia**: AE (Platia 2007), IR, OM, PA. New to KSA.

######## General distribution.

SAR.

######## Collecting month and method.

Frequent species that was collected by PT under canopies of *Acacia ehrenbergiana*, *Acacia gerrardii*, *Lycium shawii* and *Rhazya stricta*; and by LT during I-V and XI.

####### Conoderus productus arabicus

Taxon classificationAnimaliaColeopteraElateridae

(Chassain, 1979)

######## World distribution.

**Asia**: AE (Platia 2007), IR, OM, QA, SA, YE.

######## General distribution.

SAR.

######## Local distribution.

EP, MK, QS, RI (Chassain 1979 1983).

######## Collecting month and method.

A frequent species. It was collected by LT through IV-VII.

####### Heteroderes gallagheri

Taxon classificationAnimaliaColeopteraElateridae

Platia & Schimmel, 1997

######## World distribution.

**Asia**: AE (Platia 2007), IR, OM, PK. New to KSA.

######## General distribution.

SAR.

######## Collecting month and method.

Frequent species. The adults were collected by LT through IV-VI and X.

####### Heteroderus ruteri

Taxon classificationAnimaliaColeopteraElateridae

Chassain, 1979

######## World distribution.

**Asia**: OM, SA.

######## General distribution.

SAR.

######## Local distribution.

MD, MK, RI (Chassain 1979).

######## Collecting month and method.

Very rare species that was collected by PT under canopies of *Acacia gerrardii* and *Calotropis procera* during I-II.

####### Lacon modestus

Taxon classificationAnimaliaColeopteraElateridae

(Boisduval, 1835)

######## World distribution.

**Africa**: SN. **Asia**: AE (Platia 2007), ID, IL, IQ, IR, JP, LA, OM, PK, QA, SA, TW, VN, YE (Socotra). **Australia**: AU, FJ, MP, NC. **North America**: GT, US. **South America**: GF.

######## General distribution.

COS.

######## Local distribution.

EP, MK, RI (Chassain 1983).

######## Collecting month and method.

28.XII.2011

######## Collecting method.

Very rare species, which was collected by HP in XII.

####### Lanelater buettikeri

Taxon classificationAnimaliaColeopteraElateridae

Chassain, 1983

######## World distribution.

**Asia**: AE (Platia 2007), OM, SA, YE.

######## General distribution.

SAR.

######## Local distribution.

JZ, MK, NJ, RI (Chassain 1983, Platia and Schimmel 1997).

######## Collecting month and method.

Frequent species. The beetles were collected by LT during VIII-X.

###### Subfamily: Cardiophorinae

####### Craspedostethus dilutus

Taxon classificationAnimaliaColeopteraElateridae

(Erichson, 1840)

######## World distribution.

**Africa**: ER, ET, SO. **Asia**: AE, EG (Sinai), IR, OM, SA, SY, YE. **North Africa**: DZ, EG, MA. The distribution was updated from (Platia 2012).

######## General distribution.

AFR_SAR.

######## Local distribution.

BA, EP, MK (Platia and Schimmel 1997), RI (Al Dhafer et al. 2016).

######## Collecting month and method.

Abundant species that was collected by PT under canopies of *Ziziphus nummularia* and by LT during IV-IX.

####### Craspedostethus flavescens

Taxon classificationAnimaliaColeopteraElateridae

Chassain, 1979

######## World distribution.

**Asia**: AE (Platia 2007), OM, SA, YE.

######## General distribution.

SAR.

######## Local distribution.

RI (Chassain 1979).

######## Collecting month and method.

Frequent species, which was collected by LT in IV-V and VII-X.

####### Dicronychus brancuccii

Taxon classificationAnimaliaColeopteraElateridae

Platia & Schimmel, 1997

######## World distribution.

**Asia**: AE (Platia 2007), IQ, JO, KW, OM, QA, SA.

######## General distribution.

SAR.

######## Local distribution.

BA, EP, MK (Platia and Schimmel 1997).

######## Collecting month and method.

Frequent species that was collected by LT through IV-VI.

####### Dicronychus latifae

Taxon classificationAnimaliaColeopteraElateridae

Al Dhafer & Platia, 2013

######## World distribution.

**Asia**: SA.

######## General distribution.

END.

######## Local distribution.

RI (Al Dhafer and Platia 2013).

######## Collecting month and method.

Abundant species that was collected by LT during IV.

####### Dicronychus talhouki

Taxon classificationAnimaliaColeopteraElateridae

Platia & Schimmel, 1997

######## World distribution.

**Asia**: SA, YE.

######## General distribution.

SAR.

######## Local distribution.

AS, BA (Platia and Schimmel 1997), RI (Al Dhafer et al. 2016).

######## Collecting month and method.

Rare species, which was collected by PT under canopy of *Acacia gerrardii*, and by HP and LT during II-V.

####### Drasterius aegypticus

Taxon classificationAnimaliaColeopteraElateridae

Buysson, 1905

######## World distribution.

**Asia**: SA, YE. **North Africa**: DZ, EG, LY, MA, TN.

######## General distribution.

SAR.

######## Local distribution.

JZ, RI (Platia and Schimmel 1997).

######## Collecting month and method.

Rare species. It was collected by LT through III and V-VI.

##### Dermestidae

###### Subfamily: Dermestinae

####### Dermestes ater

Taxon classificationAnimaliaColeopteraDermestidae

DeGeer, 1774

######## World distribution.

**Asia**: AE, AF, AZ, CH, CN, CY, EG (Sinai), IL, IN, IQ, IR, JO, JP, KG, KP, KR, KZ, LB, MN, NP, OM, PK, RU, SA, SY, TJ, TM, TR, UZ, YE. **Europe**: AD, AL, AM, AT, BA, BE, BG, BY, CH, CZ, DE, DK, ES, FI, FR, GB, GE, GR, HR, HU, IE, IS, IT, LT, LU, LV, MK, MT, NL, NO, PL, PT, RO, RS, RU, SE, SI, SK, UA. **North Africa**: DZ, EG, ES (Canary Islands), LY, MA, PT (Madeira Archipelago), TN.

######## General distribution.

COS (Háva 2013).

######## Local distribution.

EP, RI (Mroczkowski 1979).

######## Collecting month and method.

Very rare species. It was collected by LT during III.

####### Dermestes maculatus

Taxon classificationAnimaliaColeopteraDermestidae

De Geer, 1774

######## World distribution.

**Asia**: AE, AF, AZ, CN, CY, EG (Sinai), IL, IN, IQ, IR, JO, JP, KG, KP, KR, KZ, LB, MN, NP, OM, PK, RU, SA, SY, TJ, TM, TR, UZ, YE. **Europe**: AD, AL, AM, AT, BA, BE, BG, BY, CH, CZ, DE, DK, EE, ES, FI, FR, GB, GE, GR, HR, HU, IE, IS, IT, LT, LU, LV, MK, MT, NL, NO, PL, PT, RO, RS, RU, SE, SI, SK, UA. **North Africa**: DZ, EG, ES (Canary Islands), LY, MA, PT (Madeira Archipelago), TN.

######## General distribution.

COS (Háva 2003).

######## Local distribution.

MK (Mroczkowski 1979), RI (Al Dhafer et al. 2016).

######## Collecting month and method.

Very rare species. The adult was collected by LT and PT during III and V.

###### Subfamily: Thorictinae

####### Thorictodes heydeni

Taxon classificationAnimaliaColeopteraDermestidae

Reitter, 1875

######## World distribution.

**Africa**: SD. **Asia**: CN, IL, IN, JP, PK, TR. **Australia**: AU. **Europe**: ES, FR, GB, IT, RU. **North Africa**: DZ, EG, MA. **North America**: CA, MX, US. New to Arabian Peninsula.

######## General distribution.

SCO.

######## Collecting month and method.

Very rare species. The beetles were collected by PT under the canopies of *Acacia ehrenbergiana*, *Acacia gerrardii*, *Calotropis procera* and *Lycium shawii* during IV.

####### Thorictus castaneus

Taxon classificationAnimaliaColeopteraDermestidae

Germar, 1834

######## World distribution.

**Asia**: SY. **North Africa**: DZ, EG, LY, MA. New to Arabian Peninsula.

######## General distribution.

SAR.

######## Collecting month and method.

Very rare species. It was collected by PT under the canopies of *Acacia gerrardii*, *Calotropis procera* and *Lycium shawii* during IV-VI.

####### Thorictus foreli

Taxon classificationAnimaliaColeopteraDermestidae

Wasmann, 1894

######## World distribution.

**North Africa**: DZ, MA, TN. New to Arabian Peninsula.

######## General distribution.

SAR.

######## Collecting month and method.

Very rare. The species was collected by PT under the canopy of *Acacia gerrardii* during XII.

###### Subfamily: Attageninae

####### Attagenus fasciolatus

Taxon classificationAnimaliaColeopteraDermestidae

(Solsky, 1876) 34

######## World distribution.

**Asia**: KZ, MN, SA, TJ, TM, UZ.

######## General distribution.

PAL_SAR.

######## Local distribution.

EP (Mroczkowski 1979), RI (Al Dhafer et al. 2016).

######## Collecting month and method.

A frequent species. The adults were collected by BV, SW, VC and PT on branches/under the canopies of *Acacia gerrardii*, *Calotropis procera*, *Rhazya stricta* and *Ziziphus nummularia*; and by MT through V-VI and VIII-XI.

####### Attagenus lobatus

Taxon classificationAnimaliaColeopteraDermestidae

Rosenhauer, 1856 1

######## World distribution.

**Asia**: AE, AF, CN, IN, IQ, IR, KG, KZ, MN, PK, SA, TJ, TM, TR, UZ. **Europe**: BG, CZ, ES, FR, GR, IT, RO, RU. **North Africa**: DZ, EG, MA, TN. The distribution is updated from Háva (2013).

######## General distribution.

PAL_ORR_SAR_SJP.

######## Local distribution.

EP, MK (Mroczkowski 1979), RI (Al Dhafer et al. 2016).

######## Collecting month and method.

Very rare species. It is collected by PT under canopy of *Calotropis procera* during V.

####### Attagenus posticalis

Taxon classificationAnimaliaColeopteraDermestidae

Fairmaire, 1879

######## World distribution.

**Africa**: MR, NE, SD, SN. **Asia**: AE, IL, OM, QA, SA, SY, YE. **Europe**: ES. **North Africa**: DZ, EG, MA, TN. The distribution is updated from Háva (2013).

######## General distribution.

AFR_SAR.

######## Local distribution.

EP (Háva 2011); RI (Alqarni et al. 2015; Al Dhafer et al. 2016).

######## Collecting month and method.

Frequent species. The adults were collected by BV, SW, VC and PT on branches/under canopies of *Acacia ehrenbergiana*, *Acacia gerrardii*, *Calotropis procera*, *Rhazya stricta* and *Ziziphus nummularia* through IV-X.

####### Attagenus reitteri

Taxon classificationAnimaliaColeopteraDermestidae

(Mroczkowski, 1968)

######## World distribution.

**Europe**: ES, PT. **North Africa**: DZ, MA, TN. New to Arabian Peninsula.

######## General distribution.

SAR.

######## Collecting month and method.

Very rare species that was collected by SW on branches of *Rhazya stricta* during II.

####### Attagenus scalaris

Taxon classificationAnimaliaColeopteraDermestidae

(Pic, 1893)

######## World distribution.

**North Africa**: EG, LY. New to Arabian Peninsula.

######## General distribution.

SAR.

######## Collecting month and method.

Very rare species. It was collected by SW during VI.

###### Subfamily: Megatominae

####### Anthrenus buettikeri

Taxon classificationAnimaliaColeopteraDermestidae

Mruczkowski, 1980

######## World distribution.

**Asia**: SA.

######## General distribution.

END.

######## Local distribution.

RI (Mroczkowski 1980).

######## Collecting month and method.

Very rare species. The specimens were collected by BV on branches and PT canopies of *Acacia gerrardii*, *Rhazya stricta* and *Ziziphus nummularia* during IV, VI-VII and X.

####### Anthrenus flavipes

Taxon classificationAnimaliaColeopteraDermestidae

LeConte, 1854

######## World distribution.

**Asia**: AE (Háva 2013), AF, CN, IL, IR, JP, OM, RU, SA, TJ. **Europe**: CZ, DE, DK, ES, FI, FR, GB, IT, NL. **North Africa**: EG, MA, TN.

######## General distribution.

COS (Háva 2013).

######## Local distribution.

MK, RI (Mroczkowski 1979).

######## Collecting month and method.

Very rare species that was collected by BV on branches of *Ziziphus nummularia* in IX.

####### Anthrenus malkini

Taxon classificationAnimaliaColeopteraDermestidae

Mroczkowski, 1980

######## World distribution.

**Asia**: AE, OM, QA, SA, YE (Háva 2013).

######## General distribution.

SAR.

######## Local distribution.

AS (Mroczkowski 1980).

######## Collecting month and method.

Very rare species. The beetles were collected by BV and SW on branches of *Ziziphus nummularia* in IV and IX.

####### Anthrenus verbasci

Taxon classificationAnimaliaColeopteraDermestidae

(Linnaeus, 1767)

######## World distribution.

**Asia**: AE, AF, AZ, CN, CY, EG (Sinai) IL, IN, IQ, IR, JO, JP, KG, KP, KR, KZ, LB, MN, NP, OM, PK, RU, SA, SY, TJ, TM, TR, UZ, YE. **Europe**: AD, AL, AM, AT, BA, BE, BG, BY, CH, CZ, DE, DK, EE, ES, FI, FR, GB, GE, GR, HR, HU, IE, IS, IT, LT, LU, LV, MK, MT, NL, NO, PL, PT, RO, RS, RU, SE, SI, SK, UA. **North Africa**: DZ, EG, ES (Canary Islands), LY, MA, PT (Madeira Archipelago), TN. COS.

######## General distribution.

PAL_ORR_SAR_SJP.

######## Local distribution.

RI (Alqarni et al. 2015; Al Dhafer et al. 2016).

######## Collecting month and method.

Common species that was collected by BV, VC, SW and PT on branches/under canopies of *Acacia ehrenbergiana*, *Acacia gerrardii*, *Calotropis procera*, *Rhazya stricta* and *Ziziphus nummularia*; and by LT through IV-X.

####### Phradonoma nobile

Taxon classificationAnimaliaColeopteraDermestidae

(Reitter, 1881)

######## World distribution.

**Africa**: ER, NA, SD, TJ, ZA, ZW. **Asia**: AE, AF, CY, IL, IN, IQ, IR, JO, PK, QA, SA, SY, TJ, TM, UZ. **Europe**: ES, GB, GR, PT. **North Africa**: DZ, EG, LY, MA, TN. The distribution is updated from Háva (2013).

######## General distribution.

AFR_ORR_PAL_SAR.

######## Local distribution.

AS, RI (Mroczkowski 1979).

######## Collecting month and method.

A frequent species. The specimens were collected by BV, VC, SW and PT on branches/under canopies of *Acacia ehrenbergiana*, *Acacia gerrardii*, *Calotropis procera*, *Rhazya stricta* and *Ziziphus nummularia*; and by LT through IV-X.

##### Bostrichidae

###### Subfamily: Bostrichinae

####### Enneadesmus forficula

Taxon classificationAnimaliaColeopteraBostrichidae

(Fairmaire, 1883)

######## World distribution.

**Africa**: ER, ET, SO. **Asia**: AE (Geistharde 2010), IL, IR, JO, OM, PK, SA, YE. **North Africa**: DZ, EG, LY, MA, TN.

######## General distribution.

AFR_SAR.

######## Local distribution.

MK, RI (Damoiseau 1979).

######## Collecting month and method.

Common species that was collected by BV and LT during all months except II and VII.

####### Sinoxylon senegalense

Taxon classificationAnimaliaColeopteraBostrichidae

Karsch, 1881

######## World distribution.

**Africa**: SN. **Asia**: AE (Geistharde 2010), SA, YE. **North Africa**: DZ, EG, LY, MA.

######## General distribution.

AFR_SAR.

######## Local distribution.

RI (Damoiseau 1979).

######## Collecting month and method.

Frequent species that was collected by BV and LT during all months except II, III and VIII.

###### Subfamily: Lyctinae

####### Acantholyctus cornifrons

Taxon classificationAnimaliaColeopteraBostrichidae

(Lesne, 1898)

######## World distribution.

**Africa**: DJ, ER, MZ, NA, SN, SO. **Asia**: AE (Geistharde 2010). **North Africa**: DZ, EG, MA, TN. New to KSA.

######## General distribution.

AFR_SAR.

######## Collecting month and method.

Rare species and its adults were collected by BV and LT during V, IX and X.

##### Ptinidae

###### Subfamily: Xyletininae

####### Lasioderma baudii

Taxon classificationAnimaliaColeopteraPtinidae

Schilsky, 1899

######## World distribution.

**Asia**: CY, IL, LB, SY. **Europe**: ES, FR, GR, HR, IT, PT. **North Africa**: DZ, EG, ES (Canary Islands), LY, TN. New to Arabian Peninsula.

######## General distribution.

PAL_SAR.

######## Collecting month and method.

Very rare species, which was collected by LT during IX-X.

####### Lasioderma redtenbacheri

Taxon classificationAnimaliaColeopteraPtinidae

(Bach, 1852)

######## World distribution.

**Asia**: AZ, CY, EG (Sinai), IL, IQ, KZ, MN, SY, TM, TR, UZ. **Europe**: AM, AT, BE, BG, CH, CZ, DE, ES, FR, GE, GR, HR, HU, IT, MK, NL, PL, PT, RO, RU, SK, UA. **North Africa**: EG, LY, TN. New to Arabian Peninsula.

######## General distribution.

PAL_SAR.

######## Collecting month and method.

A rare species. The adult beetles were collected by LT during IV-V and X.

###### Subfamily: Dorcatominae

####### Stagetus montanus

Taxon classificationAnimaliaColeopteraPtinidae

Toskina, 1998

######## World distribution.

**Asia**: OM, SA.

######## General distribution.

SAR.

######## Local distribution.

AS (Toskina 1998).

######## Collecting month and method.

Very rare species. It was collected by LT in V.

##### Thanerocleridae

###### Subfamily: Thaneroclerinae

####### Thaneroclerus buquet

Taxon classificationAnimaliaColeopteraThanerocleridae

(Lefebvre, 1835)

######## World distribution.

**Asia**: CN, IN, JP, RU, SA, TW. **Europe**: BE, DE, HU, NL, PL. **North Africa**: EG.

######## General distribution.

ORR_PAL_SAR_SJP.

######## Local distribution.

BA (El-Hawagry et al. 2013), RI (Al Dhafer et al. 2016).

######## Collecting month and method.

A rare species. It was collected by BV on branches and PT under canopies of *Acacia ehrenbergiana*, *Acacia gerrardii* and *Calotropis procera* during V-VII.

##### Cleridae

###### Subfamily: Tillinae

####### Eucymatodera senegalensis

Taxon classificationAnimaliaColeopteraCleridae

(Castelanu, 1832)

######## World distribution.

**Africa**: SN. **Asia**: AE, IR, OM, SA, YE. **North Africa**: DZ, EG, LY, TN. The distribution is updated from Gerstmeier (2010)

######## General distribution.

AFR_SAR.

######## Local distribution.

BA, EP, JZ, RI (Menier 1986).

######## Collecting month and method.

Frequent species that was collected by LT during V and IX-X.

####### Tillodenops plagiatus

Taxon classificationAnimaliaColeopteraCleridae

(Fairmaire, 1892)

######## World distribution.

**Africa**: KE, MR, SD, SN, SO, TZ. **Asia**: AE, IR, OM, SA, YE. The distribution is updated from Gerstmeier (2010)

######## General distribution.

AFR_SAR.

######## Local distribution.

AS, BA, JZ, MD, MK (Menier 1986).

######## Collecting month and method.

Very rare species, which was collected by LT during IV and X.

####### Wittmeridecus mediozonatus

Taxon classificationAnimaliaColeopteraCleridae

(Fairmaire, 1892)

######## World distribution.

**Africa**: DJ. **Asia**: AE, IL, OM, SA, SY, YE. **Europe**: ES, IT. **North Africa**: DZ, EG, LY, MA. The distribution is updated from Gerstmeier (2010)

######## General distribution.

AFR_PAL_SAR.

######## Local distribution.

AS, MD, MK, RI (Menier 1986).

######## Collecting month and method.

A frequent species. The adults were collected by VC on branches of *Acacia ehrenbergiana* and by LT through IV-X.

###### Subfamily: Korynetinae

####### Necrobia rufipes

Taxon classificationAnimaliaColeopteraCleridae

(De Geer, 1775)

######## World distribution.

**Asia**: AE, AZ, CN, IN, IR, JP, MN, OM, RU, SA, TJ, TR. **Europe**: AM, AT, BE, BY, CH, CZ, DE, DK, EE, ES, FI, FR, GB, GR, HR, HU, IE, IT, LI, LT, LV, NL, PT, RO, RU, SE, SI, SK. **North Africa**: DZ, EG, ES (Canary Islands), LY, MA, TN.

######## General distribution.

COS (Gerstmeier 2010)

######## Local distribution.

BA (El-Hawagry et al. 2013), EP, MK, RI (Menier 1986).

######## Collecting month and method.

Frequent species, which was collected by VC on branches of *Lycium shawii* and by LT during IV-VIII and X.

###### Melyridae

####### Subfamily: Dasytinae

######## Danacea sp.

Taxon classificationAnimaliaColeopteraCleridae

######### Collecting month and method.

Frequent species. The specimens were collected by BV, SW and PT on branches/under canopies of *Acacia ehrenbergiana*, *Acacia gerrardii*, *Calotropis procera*, *Lycium shawii*, *Rhazya stricta* and *Ziziphus nummularia*; and by HP and LT during II-V and XII.

####### Subfamily: Malachinae

######## Colotes javeti

Taxon classificationAnimaliaColeopteraCleridae

Du Val, 1852

######### World distribution.

**Europe**: ES, FR, IT, PT. **North Africa**: DZ, EG, MA, TN. New to Arabian Peninsula.

######### General distribution.

PAL_SAR.

######### Collecting month and method.

A frequent species. The beetles were collected by BV, SW, VC and PT on branches/under canopies of *Acacia ehrenbergiana*, *Acacia gerrardii*, *Calotropis procera*, *Lycium shawii*, *Rhazya stricta* and *Ziziphus nummularia*; and by HP during II-VI and IX.

######## Malachius sp.1

Taxon classificationAnimaliaColeopteraCleridae

######### Collecting month and method.

A common species. The adults were collected by BV, SW, VC and PT on branches/under canopies of *Acacia ehrenbergiana*, *Acacia gerrardii*, *Calotropis procera*, *Lycium shawii*, *Rhazya stricta* and *Ziziphus nummularia*; and by HP and LT through V-IX.

######## Malachius sp.2

Taxon classificationAnimaliaColeopteraCleridae

######### Collecting month and method.

A rare species. It was collected by BV on branches of *Acacia gerrardii* and *Ziziphus nummularia*, by PT under canopies of *Acacia ehrenbergiana* and *Calotropis procera*; and by HP in III-IV and IX.

###### Cryptophagidae

####### Subfamily: Cryptophaginae

######## Cryptophagus acutangulus

Taxon classificationAnimaliaColeopteraCleridae

Gyllenhal, 1827

######### World distribution.

**Africa**: sub-saharan Africa. **Asia**: AF, AZ, CN, EG (Sinai), IR, JP, KG, KP, KZ, PK, RU, SA, TJ, TM, UZ. **Europe**: AT, BA, BE, BY, CH, CZ, DE, DK, EE, FI, FR, GB, GE, GR, HU, IE, IS, IT, LT, LV, NL, NO, PL, RO, RS, RU, SE, SI, SK, UA. **North Africa**: EG, MA. **North America**: CA, MX, US.

######### General distribution.

COS (Otero 2013).

######### Local distribution.

EP (Johnson 1989), RI (Al Dhafer et al. 2016).

######### Collecting month and method.

Frequent species. The adults were collected by BV on branches and PT under canopies of *Acacia ehrenbergiana*, *Acacia gerrardii*, *Calotropis procera*, *Lycium shawii*, *Rhazya stricta* and *Ziziphus nummularia*; and by LT during I-III, V, VIII and XI-XII.

####### Subfamily: Atomariinae

######## Atomaria fuscata

Taxon classificationAnimaliaColeopteraCleridae

(Schönherr, 1808)

######### World distribution.

**Asia**: AZ, CN, IL, JP, RU. **Europe**: AM, AT, BA, BE, BY, CH, CZ, DE, DK, EE, ES, FI, FR, GB, GE, GR, HR, HU, IE, IT, LI, LT, LV, NL, NO, PL, RO, RU, SE, SI, SK, UA. **North America**: CA. New to Arabian Peninsula.

######### General distribution.

NAR_PAL_SAR_SJP.

######### Collecting month and method.

Very rare species that was collected by LT in X.

###### Phalacridae

####### Subfamily: Phalacrinae

######## Olibrosoma testacea

Taxon classificationAnimaliaColeopteraCleridae

Tournier, 1889

######### World distribution.

**Asia**: AE (Švec 2010b), JO, SA. **Europe**: ES. **North Africa**: DZ, EG, MA, TN.

######### General distribution.

SAR.

######### Local distribution.

RI (Al Dhafer et al. 2016).

######### Collecting month and method.

Afrequent species. The adults were collected by PT under canoppy of *Acacia ehrenbergiana*, and by LT during IV-X.

###### Laemophloeidae

####### Subfamily: Laemophloeinae

######## Placonotus testaceus

Taxon classificationAnimaliaColeopteraCleridae

(Fabricus, 1787)

######### World distribution.

**Asia**: AZ, BT, CN, IN, JP, KG, KZ, RU, TW, UZ. **Australia**: AU. **Europe**: AM, BE, CH, CZ, DE, DK, ES, FR, GE, IT, PL, PT, SK. **North Africa**: DZ, TN. New to Arabian Peninsula.

######### General distribution.

SCO.

######### Collecting month and method.

Very rare species that was collected by LT during VIII and XI.

###### Nitidulidae

####### Subfamily: Carpophilinae

######## Carpophilus hemipterus

Taxon classificationAnimaliaColeopteraCleridae

(Linnaeus, 1758)

######### World distribution.

**Africa**: MR, ZA. **Asia**: AE, AZ, CN, IL, IN, IQ, IR, JO, JP, LB, SA, TM, TR, TW. **Europe**: AL, AT, BA, BE, BY, CH, CZ, DE, DK, ES, FI, FR, GB, GR, HR, HU, IE, IS, IT, LV, MD, MT, NL, PL, PT, SE, SK. **North Africa**: DZ, EG, ES (Canary Islands), LY, PT (Madeira Archipelago), TN. **North America**: GT, MX, NI, PA, US.

######### General distribution.

COS (Jelinek 1979).

######### Local distribution.

EP, MK, RI (Beccari 1971, Jelinek 1979 1988).

######### Collecting month and method.

Rare species. The adult beetles were collected by PT under canoppy of *Rhazya stricta*, and by HP and LT during IV-V and XI.

####### Subfamily: Nitidulinae

######## Nitidula eremita

Taxon classificationAnimaliaColeopteraCleridae

Audisio, 1990

######### World distribution.

**Asia**: AE, IL, IQ, IR, SA. **North Africa**: DZ, EG, LY, TN.

######### General distribution.

SAR.

######### Local distribution.

EP, RI, (Jelinek 1979 1988).

######### Collecting month and method.

Frequent species that was collected by PT under canoppy of *Lycium shawii* during I, and by LT during III and X-XII.

####### Subfamily: Cybocephalinae

######## Cybocephalus rufifrons flaviceps

Taxon classificationAnimaliaColeopteraCleridae

Reitter, 1874

######### World distribution.

**Asia**: IQ, IR, SA, TR. **North Africa**: DZ, EG, MA, TN.

######### General distribution.

SAR.

######### Local distribution.

MK (Endrödy-Younga 1979), RI (Beccari 1971).

######### Collecting month and method.

A common species. The specimens were collected by BV, SW and VC on branches of *Acacia ehrenbergiana*, *Acacia gerrardii*, *Calotropis procera*, *Lycium shawii*, *Rhazya stricta* and *Ziziphus nummularia*; and by MT through II, V-IX and XII.

###### Coccinellidae

####### Subfamily: Coccinellinae

######## Bulaea lividula bocandei

Taxon classificationAnimaliaColeopteraCleridae

Mulsant, 1850

######### World distribution.

**Africa**: ER. **Asia**: AE, IL, IQ, IR, JO, PK, SA, SY, YE. **North Africa**: DZ, EG, MA.

######### General distribution.

AFR_SAR.

######### Local distribution.

EP (Fürsch 1979).

######### Collecting month and method.

Very rare species that was collected by BV on branches of *Ziziphus nummularia* in VIII.

######## Coccinella septempunctata

Taxon classificationAnimaliaColeopteraCleridae

Linnaeus, 1758

######### World distribution.

**Africa**: ZA. **Asia**: AF, AZ, BT, CN, CY, EG (Sinai), IL, IN, IQ, IR, JO, JP, KG, KP, KR, KW, KZ, LB, MN, NP, PK, RU, SA, SY, TJ, TM, TR, TW, UZ. **Europe**: AD, AL, AM, AT, BA, BE, BG, BY, CH, CZ, DE, DK, EE, ES, FI, FR, GB, GE, GR, HR, HU, IE, IT, LI, LT, LU, LV, MD, MK, NL, NO, PL, PT, RO, RS, RU, SE, SI, SK, UA. **North Africa**: PT (Madeira Archipelago). **North America**: CA, US.

######### General distribution.

SCO.

######### Local distribution.

RI (Fürsch 1979).

######### Collecting month and method.

Very rare species, which was collected by SW during IV.

######## Coccinella undecimpunctata menetriesi

Taxon classificationAnimaliaColeopteraCleridae

Mulsant, 1850

######### World distribution.

**Asia**: AE, AF, CN, EG (Sinai), IL, IN, IQ, IR, JO, KG, KW, KZ, MN, PK, RU, SA, SY, TR. **Europe**: GR, IT, PT, RU. **North Africa**: DZ, EG, LY, TN.

######### General distribution.

PAL_ORR_SAR_SJP.

######### Local distribution.

RI (Fürsch 1979).

######### Collecting month and method.

A frequent species. The adult beetles were collected by BV, SW, VC and PT on branches/under canopies of *Acacia ehrenbergiana, Acacia gerrardii*, *Calotropis procera*, *Lycium shawii*, *Rhazya stricta* and *Ziziphus nummularia* during I-IV and XI.

######## Diomus rubidus

Taxon classificationAnimaliaColeopteraCleridae

(Motschulsky, 1837)

######### World distribution.

**Asia**: IL, IQ, IR, LB, SA, SY, YE. **Europe**: AM, FR, GR, HR, IT. **North Africa**: DZ, EG, LY, MA, TN.

######### General distribution.

PAL_SAR.

######### Local distribution.

RI (Fürsch 1979).

######### Collecting month and method.

Frequent species. The specimens were collected by BV and SW on branches and PT under canopies of *Acacia ehrenbergiana*, *Acacia gerrardii*, *Calotropis procera*, *Lycium shawii*, and *Rhazya stricta* through I-VII.

######## Hippodamia variegata

Taxon classificationAnimaliaColeopteraCleridae

(Goeze, 1777)

######### World distribution.

**Africa**: SZ, ZA. **Asia**: AE, AF, AZ, BT, CN, EG (Sinai), IL, IN, IQ, IR, JO, KG, KP, KR, KZ, LB, MN, NP, PK, RU, SA, SY, TJ, TM, TR, UZ, YE. **Europe**: AD, AL, AM, AT, BA, BE, BG, BY, CH, CZ, DE, DK, EE, ES, FI, FR, GB, GE, GR, HR, HU, IT, LI, LT, LU, LV, MD, MK, NL, PL, PT, RO, RS, RU, SE, SI, SK, UA. **North Africa**: DZ, EG, ES (Canary Islands), LY, MA, PT (Madeira Archipelago), TN. **North America**: US.

######### General distribution.

COS.

######### Local distribution.

BA (El-Hawagry et al. 2013), JZ (Beccari 1971), RI (Talhouk 1982; Al Dhafer et al. 2016).

######### Collecting month and method.

Frequent species. The specimens were collected by BV, SW, VC and PT on branches/ under canopies of *Acacia gerrardii*, *Calotropis procera*, *Lycium shawii*, *Rhazya stricta* and *Ziziphus nummularia*; and by LT and MT in I and III-IV.

######## Hyperaspis vinciguerrae

Taxon classificationAnimaliaColeopteraCleridae

Capra, 1929

######### World distribution.

**Africa**: GM, SN. **Asia**: AE (Raimundo et al. 2007), SA, YE. **North Africa**: LY.

######### General distribution.

AFR_SAR.

######### Local distribution.

RI (Talhouk 1982)

######### Collecting month and method.

Rare species that was collected by BV, SW and VC on branches of *Calotropis procera*, *Lycium shawii*, *Rhazya stricta* and *Ziziphus nummularia*; and MT during V-VIII.

######## Nephus arcuatus

Taxon classificationAnimaliaColeopteraCleridae

Kapur, 1959

######### World distribution.

**Africa**: TG. **Asia**: AE (Raimundo et al. 2007), IR, SA, YE.

######### General distribution.

AFR_SAR.

######### Local distribution.

AS (Fürsch 1979).

######### Collecting month and method.

Very rare species, which was collected by VC on branches of *Ziziphus nummularia* in VII.

######## Nephus levaillanti

Taxon classificationAnimaliaColeopteraCleridae

(Mulsant, 1850)

######### World distribution.

**Africa**: ZA. **Asia**: AF, CN, IL, IN, IR, JO, JP, LB, PK, RU, SA, TW, YE. **Europe**: GR, IT. **North Africa**: EG.

######### General distribution.

AFR_ORR_PAL_SAR_SJP.

######### Local distribution.

RI (Al Dhafer et al. 2016).

######### Collecting month and method.

Very rare species. The specimens were collected by BV on branches of *Acacia gerrardii* and *Ziziphus nummularia*; and by PT under canopies of *Acacia ehrenbergiana* and *Acacia gerrardii* during IV and VII.

######## Nephus wittmeri

Taxon classificationAnimaliaColeopteraCleridae

Fürsch, 1979

######### World distribution.

**Asia**: SA, YE.

######### General distribution.

SAR.

######### Local distribution.

RI (Fürsch 1979).

######### Collecting month and method.

Rare species. The adults were collected by BV on branches of *Acacia ehrenbergianaAcacia gerrardii* and *Ziziphus nummularia*; by PT under canopy of *Acacia ehrenbergiana*; and by VC on branches of *Rhazya stricta* during II-V and XII.

######## Parexochomus pubescens

Taxon classificationAnimaliaColeopteraCleridae

(Küster, 1848)

######### World distribution.

**Africa**: Africa. **Asia**: AE (Raimundo et al. 2007), AF, IL, IN, IQ, IR, SA, SY, YE. **Europe**: ES, FR, GR, IT. **North Africa**: DZ, EG, LY, MA, TN.

######### General distribution.

AFR_ORR_PAL_SAR

######### Local distribution.

AS, RI (Fürsch 1979).

######### Collecting month and method.

Common beetles that were collected by BV and VC on branches of *Acacia ehrenbergianaAcacia gerrardii*, *Calotropis procera* and *Lycium shawii* throughout the year except in VI and VIII.

######## Scymnus luxorensis

Taxon classificationAnimaliaColeopteraCleridae

Fürsch, 1989

######### World distribution.

**Asia**: SA. **North Africa**: EG.

######### General distribution.

SAR.

######### Local distribution.

RI (Fürsch 1989).

######### Collecting month and method.

Very rare species that was collected by SW on branches of *Rhazya stricta* during XII.

######## Scymnus nubilus

Taxon classificationAnimaliaColeopteraCleridae

Mulsant, 1850

######### World distribution.

**Africa**: KE, SZ, UG. **Asia**: AE (Raimundo et al. 2007), AF, BD, IL, IN, IQ, IR, JO, JP, KW, LB, NP, OM, PK, SA, SY, TR, TW, YE. **Australia**: AU. **Europe**: ES, GR, IT, PT. **North Africa**: EG, ES (Canary Islands), PT (Madeira Archipelago).

######### General distribution.

SCO.

######### Local distribution.

AS (Fürsch 1979; Raimundo et al 2006); RI (Al Dhafer et al. 2016).

######### Collecting month and method.

A rare species, which was collected by PT under canopy of *Lycium shawii*, and by LT and MT during V and VII-VIII.

######## Scymnus subvillosus

Taxon classificationAnimaliaColeopteraCleridae

(Goeze, 1777)

######### World distribution.

**Africa**: ZA. **Asia**: AE, AF, AZ, CY, EG (Sinai), IL, IQ, IR, JO, KG, KW, KZ, LB, PK, QA, SA, SY, TJ, TR, UZ, YE. **Europe**: AD, AL, AM, AT, BA, BG, CH, CZ, DE, ES, FR, GE, GR, HR, HU, IT, MK, PT, RO, RS, RU, SI, SK, UA. **North Africa**: DZ, EG, ES (Canary Islands), LY, MA, PT (Madeira Archipelago), TN.

######### General distribution.

AFR_PAL_SAR.

######### Local distribution.

AS, RI (Fürsch 1979).

######### Collecting month and method.

Common species. The adults were collected by BV, SW, VC and PT on branches/under canopies of *Acacia ehrenbergianaAcacia gerrardii*, *Calotropis procera*, *Lycium shawii*, *Rhazya stricta* and *Ziziphus nummularia* throughout the year except in X.

######## Scymnus syriacus

Taxon classificationAnimaliaColeopteraCleridae

(Marseul, 1868)

######### World distribution.

**Asia**: CY, EG (Sinai), IL, IQ, IR, JO, LB, SA, SY. **North Africa**: EG.

######### General distribution.

SAR.

######### Local distribution.

BA (El-Hawagry et al. 2013), EP (Fürsch 1989).

######### Collecting month and method.

A common species that was collected by BV, SW and VC on branches of *Acacia ehrenbergianaAcacia gerrardii*, *Calotropis procera*, *Lycium shawii*, *Rhazya stricta* and *Ziziphus nummularia*; and by HP during II-VII and X-XII.

######## Scymnus yemenensis

Taxon classificationAnimaliaColeopteraCleridae

(Kapur, 1959)

######### World distribution.

**Asia**: AE (Raimundo et al. 2007), OM, SA, YE.

######### General distribution.

SAR.

######### Local distribution.

AS, RI (Fürsch 1979).

######### Collecting month and method.

A rare species that was collected by BV on branches of *Acacia ehrenbergiana*, by SW and VC on branches of *Calotropis procera*, and by LT during IV and X.

###### Mycetophagidae

####### Subfamily: Mycetophaginae

######## Typhaea stercorea

Taxon classificationAnimaliaColeopteraCleridae

(Linnaeus, 1758)

######### World distribution.

**Africa**: GM, MR, ZA. **Asia**: AE, AF, AZ, BT, CN, CY, EG (Sinai), IL, IQ, IR, JO, JP, KG, KR, KZ, MN, NP, PK, RU, SA, SY, TJ, TM, TR, UZ, YE. **Europe**: AD, AL, AM, AT, BA, BE, BG, BY, CH, CZ, DE, DK, EE, ES, FI, FO, FR, GB, GE, GR, HR, HU, IE, IS, IT, LI, LT, LU, LV, MD, MK, MT, NL, NO, PL, PT, RO, RS, RU, SE, SI, SK, UA. **North Africa**: DZ, EG, ES (Canary Islands), LY, MA, PT (Madeira Archipelago), TN. **South America**: CL.

######### General distribution.

COS.

######### Local distribution.

BA (El-Hawagry et al. 2013).

######### Collecting month and method.

Very rare species. The adults were collected by LT during V.

###### Tenebrionidae

####### Subfamily: Lagriinae

######## Centorus csikii bagdadensis

Taxon classificationAnimaliaColeopteraCleridae

(Reitter, 1920)

######### World distribution.

**Asia**: CY, IL, IQ, JO, SA, YE.

######### General distribution.

SAR.

######### Local distribution.

EP, RI (Kaszab 1979).

######### Collecting month and method.

Common beetles that were collected by PT under the canopies *Acacia ehrenbergiana*, *Acacia gerrardii* and *Ziziphus nummularia*; and by LT throughout the year except in XI.

####### Subfamily: Pimeliinae

######## Adelostoma subtile

Taxon classificationAnimaliaColeopteraCleridae

Reitter, 1900

######### World distribution.

**Asia**: IL, IR, JO, SA, SY, TR.

######### General distribution.

PAL_SAR.

######### Local distribution.

MD, NJ, RI (Kaszab 1981 1982).

######### Collecting month and method.

A frequent species. The adults were collected by PT under the canopies *Acacia ehrenbergiana*, *Acacia gerrardii*, *Rhazya stricta* and *Ziziphus nummularia*; and by HP through II, IV-V, and VII-X.

######## Adesmia cancellata

Taxon classificationAnimaliaColeopteraCleridae

(Klug, 1830)

######### World distribution.

**Asia**: AE, BH, EG (Sinai), IL, IR, IQ, JO, KW, OM, PK, SA, SY, YE. The distribution is updated from Schawaller (2010).

######### General distribution.

SAR.

######### Local distribution.

AS, BA, EP, JZ, MD, MK, NJ, RI (Kaszab 1979 1981 1982; El-Hawagry et al. 2013).

######### Collecting month and method.

Very rare species. It was collected by PT under canopy of *Acacia ehrenbergiana* and *Lycium shawii* in III and V.

######## Akis spinosa

Taxon classificationAnimaliaColeopteraCleridae

(Linnaeus, 1764)

######### World distribution.

**North Africa**: EG. New to Arabian Peninsula,

######### General distribution.

SAR.

######### Collecting month and method.

Very rare species that was collected by PT under canopy of *Lycium shawii* during VII.

######## Akis subtricostata

Taxon classificationAnimaliaColeopteraCleridae

Redtenbacher, 1850

######### World distribution.

**Asia**: AE (Schawaller 2010), IQ, IR, SY. New to KSA.

######### General distribution.

SAR.

######### Collecting month and method.

Very rare species, which was collected by LT in IX.

######## Ammogiton sonyae

Taxon classificationAnimaliaColeopteraCleridae

Kaszab, 1979

######### World distribution.

**Asia**: SA.

######### General distribution.

END.

######### Local distribution.

EP, RI (Kaszab 1979 1981 1982).

######### Collecting month and method.

Very rare species that was collected by HP during II.

######## Apentanodes arabicus

Taxon classificationAnimaliaColeopteraCleridae

(Kirchsberg, 1877)

######### World distribution.

**Asia**: AE, OM, SA.

######### General distribution.

SAR.

######### Local distribution.

AS, EP, HA, MD, MK, QS, RI, (Kaszab 1979 1981 1982).

######### Collecting month and method.

Common beetles that were collected by PT under canopies of *Acacia ehrenbergiana*, *Acacia gerrardii*, *Calotropis procera*, *Lycium shawii*, *Rhazya stricta* and *Ziziphus nummularia*; and by HP and LT through I-VI.

######## Boromorphus saudicus

Taxon classificationAnimaliaColeopteraCleridae

Schawaller, Al Dhafer & Fadl, 2013

######### World distribution.

**Asia**: SA (Schawaller et al. 2013).

######### General distribution.

END.

######### Local distribution.

RI (Schawaller et al. 2013).

######### Collecting month and method.

A rare beetle. The adults were collected by PT under canopies of *Acacia ehrenbergiana*, *Acacia gerrardii*, *Calotropis procera*, *Lycium shawii* and *Ziziphus nummularia*; and by HP during II-IV.

######## Cyphostethe ferruginea

Taxon classificationAnimaliaColeopteraCleridae

(Marseul, 1867)

######### World distribution.

**Asia**: AE, IL, SA. **North Africa**: DZ, LY, TN.

######### General distribution.

SAR.

######### Local distribution.

AS, RI (Kaszab 1981).

######### Collecting month and method.

Very rare species that was collected by PT under canopy of *Calotropis procera* in X.

######## Cyphostethe wittmeri

Taxon classificationAnimaliaColeopteraCleridae

Kaszab, 1979

######### World distribution.

**Asia**: AE, SA.

######### General distribution.

SAR.

######### Local distribution.

EP, HA, MK, RI (Kaszab 1979 1981 1982).

######### Collecting month and method.

A rare species. The specimens were collected by LT through V-IX.

######## Erodius glabratus

Taxon classificationAnimaliaColeopteraCleridae

Solier, 1834

######### World distribution.

**Asia**: EG (Sinai), SA. **North Africa**: EG.

######### General distribution.

SAR.

######### Local distribution.

AS, EP, HA, JF, JZ, MD, MK, QS, TB, (Kaszab 1979 1981 1982).

######### Collecting month and method.

Very rare species that was collected by LT in IV.

######## Erodius octocostatus

Taxon classificationAnimaliaColeopteraCleridae

Peyerimhoff, 1907

######### World distribution.

**Asia**: EG (Sinai), IQ, JO, SA. **North Africa**: EG.

######### General distribution.

SAR.

######### Local distribution.

ES, NJ (Kaszab 1981).

######### Collecting month and method.

Very rare species. The beetles were collected by HP during II and XII.

######## Erodius servillei

Taxon classificationAnimaliaColeopteraCleridae

Solier, 1834

######### World distribution.

**Asia**: AF, IQ, IR, SA, SY.

######### General distribution.

SAR.

######### Local distribution.

MK, QS, RI (Kaszab 1979 1981)

######### Collecting month and method.

Very rare species that was collected by PT under canopy of *Rhazya stricta* through III.

######## Mesostena angustata

Taxon classificationAnimaliaColeopteraCleridae

(Fabricius, 1775)

######### World distribution.

**Africa**: ER, NG, SD. **Asia**: EG (Sinai), IL, IQ, IR, JO, LB, SA (Al Dhafer et al. 2016), SY. **North Africa**: DZ, EG, LY, TN.

######### General distribution.

AFR_SAR.

######### Local distribution.

RI (Al Dhafer et al. 2016).

######### Collecting month and method.

Very rare species. The specimens were collected by PT under canopy of *Acacia gerrardii* through V; and by HP in V and XII.

######## Mesostena puncticollis

Taxon classificationAnimaliaColeopteraCleridae

Solier, 1835

######### World distribution.

**Africa**: SD, SO. **Asia**: AE, EG (Sinai), IL, IQ, IR, JO, SA, SY, TM, YE. **Europe**: AM. **North Africa**: EG.

######### General distribution.

AFR_PAL_SAR.

######### Local distribution.

AS, EP, HA, MD, MK, NJ, QS, RI, TB (Kaszab 1979 1981 1982).

######### Collecting month and method.

An abundant species. The adults of this beetle were collected by HP, LT and PT all over the year except in XI.

######## Oxycara saudarabica

Taxon classificationAnimaliaColeopteraCleridae

Kaszab, 1979 

######### World distribution.

**Asia**: AE, SA.

######### General distribution.

SAR.

######### Local distribution.

EP, RI (Kaszab 1979 1981 1982).

######### Collecting month and method.

Common species that was collected by HP, LT and PT throughout the year.

######## Paraplatyope arabica arabica

Taxon classificationAnimaliaColeopteraCleridae

(Blair, 1931)

######### World distribution.

**Asia**: AE, KW, SA.

######### General distribution.

SAR.

######### Local distribution.

EP, MK, RI (Kaszab 1979 1982).

######### Collecting month and method.

Very rare species that was collected by LT during III.

######## Pimelia arabica

Taxon classificationAnimaliaColeopteraCleridae

(Klug, 1830)

######### World distribution.

**Asia**: AE, BH, IL, IQ, JO, KW, OM, PK, SA, SY, YE. **North Africa**: EG.

######### General distribution.

SAR.

######### Local distribution.

AS, BA, EP, HA, MD, MK, RI (Kaszab 1979 1982)

######### Collecting month and method.

A rare species. The beetles were collected by HP and PT in X-XII.

######## Pimelia longula

Taxon classificationAnimaliaColeopteraCleridae

Kwieton, 1981

######### World distribution.

**Asia**: BH, SA.

######### General distribution.

SAR.

######### Local distribution.

EP, MD, RI, TB (Kaszab 1979 1982).

######### Collecting month and method.

Very rare species that was collected by HP during I.

######## Prionotheca coronata ovalis

Taxon classificationAnimaliaColeopteraCleridae

Ancey, 1881

######### World distribution.

**Asia**: AE, BH, IQ, IR, SA, YE.

######### General distribution.

SAR.

######### Local distribution.

AS, EP, HA, MD, MK, QS, RI (Kaszab 1979 1982).

######### Collecting month and method.

A rare species. This beetle was collected by HP, LT and PT during II, IV, VI and X.

######## Thriptera kraatzi

Taxon classificationAnimaliaColeopteraCleridae

Haag-Rutenberg, 1876 

######### World distribution.

**Asia**: AE, EG (Sinai), IN, IR, JO, OM, PK, SA, YE. **North Africa**: AG.

######### General distribution.

ORR_SAR.

######### Local distribution.

AS, BA, MK, RI (Kaszab 1979 1982; El-Hawagry et al. 2013).

######### Collecting month and method.

A frequent species. The specimens were collected by HP and PT during III-VI and VIII.

######## Trachyderma philistina

Taxon classificationAnimaliaColeopteraCleridae

Reiche & Saulcy, 1857

######### World distribution.

**Asia**: AE, BH, CY, EG (Sinai), IL, IQ, IR, JO, OM, SA, SY, TR, YE. **Europe**: GR. **North Africa**: EG. According to the Palaearctic catalogue *Trachyderma philistina* occurs east of the Arabian Peninsula also in Iran and in the ORR region (Löbl et al. 2008). However, the known distribution of species indicates its absence from Afghanistan, Pakistan, and India. The only record available from western India apparently is by Kaszab (1982). This record is a misidentification and Kaszab (1982) did not specifically mention this species from Afghanistan (W. Schawaller, personal communication, February 29 2016).

######### General distribution.

PAL_SAR.

######### Local distribution.

EP, HA, MK, NJ, QS, RI, TB (Kaszab 1979 1982)

######### Collecting month and method.

Rare species that was collected by HP and PT through III-VI, X and XII.

######## Trichosphaena arabica

Taxon classificationAnimaliaColeopteraCleridae

Kaszab, 1961

######### World distribution.

**Asia**: AE, OM, SA, YE.

######### General distribution.

SAR.

######### Local distribution.

HA, JZ, MK, NJ, QS, RI (Kaszab 1979 1981 1982).

######### Collecting month and method.

A frequent species. The adults of this species were collected by BV on branches of *Acacia ehrenbergiana* and *Ziziphus nummularia*, and by HP during IV and VII-IX.

######## Zophosis punctata

Taxon classificationAnimaliaColeopteraCleridae

Brullé, 1832

######### World distribution.

**Asia**: AF, AZ, CN, CY, EG (Sinai), IL, IQ, IR, JO, KZ, LB, PK, SA, SY, TJ, TM, TR, UZ. **Europe**: AL, AM, ES, GR, IT. **North Africa**: DZ, LY, MA, TN.

######### General distribution.

PAL_SAR_SJP.

######### Local distribution.

MK, QS (Kaszab 1981 1982), RI (Al Dhafer et al. 2016)

######### Collecting month and method.

Frequent species. The adults were collected by HP and PT through II-IV and VI-X.

####### Subfamily: Tenebrioninae

######## Alphitobius laevigatus

Taxon classificationAnimaliaColeopteraCleridae

(Fabricius, 1781)

######### World distribution.

**Africa**: GH. **Asia**: AE, AF, BH, BT, CN, CY, IN, IQ, IR, JP, KZ, RU, SA, TW, YE. **Europe**: AM, BE, CH, DE, ES, GB, GR, HU, IT, LU, MT, NL, PL, PT, RU, SK, UA. **North Africa**: EG, ES (Canary Islands), LY, PT (Madeira Archipelago), TN. **North America**: MX, US.

######### General distribution.

COS (Schawaller 2010).

######### Local distribution.

EP, MD, MK, RI (Kaszab 1979 1982).

######### Collecting month and method.

Very rare species that was collected by LT during X.

######## Blaps kollari

Taxon classificationAnimaliaColeopteraCleridae

Seidlitz, 1893

######### World distribution.

**Asia**: AE, IQ, JO, OM, SA, YE. **North Africa**: EG.

######### General distribution.

SAR.

######### Local distribution.

AS, BA, EP, HA, MD, MK, QS, RI (Kaszab 1979 1981 1982; El-Hawagry et al. 2013).

######### Collecting month and method.

A frequent species. The adult beetles were collected by HP, LT and PT during I-II, V-VI and X-XII.

######## Cheirodes brevicollis

Taxon classificationAnimaliaColeopteraCleridae

(Wollaston, 1864)

######### World distribution.

**Africa**: SO. **Asia**: AE, AF, BH, CN, EG (Sinai), IL, IQ, IR, JO, KZ, MN, OM, PK, SA, TM, UZ, YE. **Europe**: ES, IT, MT. **North Africa**: DZ, EG, ES (Canary Isalands), LY, MA, TN.

######### General distribution.

AFR_PAL_SAR_SJP.

######### Local distribution.

AS, EP, HA, JZ, MD, MK, RI (Kaszab 1979 1982). It is reported as *Anemia brevicollis* (Wollaston 1864) from BA (El-Hawagry et al. 2013).

######### Collecting month and method.

Common species. The specimens were collected by LT and PT through IV-X.

######## Cheirodes pilosus

Taxon classificationAnimaliaColeopteraCleridae

(Tournier, 1868)

######### World distribution.

**Africa**: SD, TD. **Asia**: AE (Schawaller 2010), EG (Sinai), IL, OM, SA, YE. **North Africa**: DZ, EG, ES (Canary Isalands), LY, MA, TN.

######### General distribution.

AFR_SAR.

######### Local distribution.

EP, HA, MD, MK, QS, RI (Kaszab 1979 1982).

######### Collecting month and method.

A rare species. The adults were collected by BV and SW on branches of *Lycium shawii* and *Calotropis procera*, respectively during VII; and by LT in V-VI.

######## Cheirodes sardous

Taxon classificationAnimaliaColeopteraCleridae

(Gené, 1839)

######### World distribution.

**Asia**: AE, AZ, CY, EG (Sinai), IL, IQ, IR, JO, SA, TR. **Australia**: AU. **Europe**: AM, ES, FR, GR, IT, PT. **North Africa**: EG, ES (Canary Isalands), LY, MA, TN.

######### General distribution.

AUS_PAL_SAR.

######### Local distribution.

AS, EP, HA, MD, MK, NJ, RI, TB (Kaszab 1979 1982).

######### Collecting month and method.

Very rare species that was collected by LT during VII.

######## Gonocephalum prolixum

Taxon classificationAnimaliaColeopteraCleridae

(Erichson, 1843)

######### World distribution.

**Africa**: ZA. **Asia**: AE, CY, IR, SA, SY. **Europe**: ES, IT. **North Africa**: DZ, EG, ES (Canary Isalands), LY, MA, TN.

######### General distribution.

AFR_PAL_SAR.

######### Local distribution.

AS, MD, MK (Kaszab 1979 1982), RI (Al Dhafer et al. 2016).

######### Collecting month and method.

An abundant species. The beetles were collected by HP, LT and PT throughout the year.

######## Gonocephalum rusticum

Taxon classificationAnimaliaColeopteraCleridae

(Olivier, 1811)

######### World distribution.

**Asia**: AF, AZ, CN, EG (Sinai), IL, IQ, IR, KZ, MN, OM, RU, SA, TJ, TM, TR, UZ. **Europe**: AL, AM, ES, FR, GR, HR, IT, MT, MK, PT, RU, UA. **North Africa**: EG, ES (Canary Isalands), LY, MA, PT (Madeira Archipelago), TN.

######### General distribution.

PAL_SAR_SJP.

######### Local distribution.

AS, EP, MK, RI (Kaszab 1979 1982).

######### Collecting month and method.

Common species that was collected by HP, LT and PT all over the year.

######## Gonocephalum setulosum

Taxon classificationAnimaliaColeopteraCleridae

(Faldermann, 1837)

######### World distribution.

**Africa**: ZA. **Asia**: AE (Schawaller 2010), AF, CN, CY, EG (Sinai), IL, IQ, IR, KZ, LB, SA, SY, TJ, TM, TR, UZ, YE. **Europe**: AM, ES, GR, IT, MT, RU. **North Africa**: EG, ES (Canary Isalands), LY, MA, TN.

######### General distribution.

AFR_PAL_SAR_SJP.

######### Local distribution.

EP, HA, MD, MK, QS, RI, TB (Kaszab 1979 1982).

######### Collecting month and method.

Common species that was collected by HP, LT and PT through II, IV-X and XII.

######## Gonocephalum soricinum

Taxon classificationAnimaliaColeopteraCleridae

(Reiche & Saulcy, 1857)

######### World distribution.

**Africa**: ET. **Asia**: AE (Schawaller 2010), AF, EG (Sinai), IL, IR, JO, PK, SA, SY, YE.

######### General distribution.

AFR_SAR.

######### Local distribution.

AS, BA, HA, MK, NJ, RI (Kaszab 1979 1982).

######### Collecting month and method.

Rare species that was collected by HP, LT and PT in I, V and IX-X.

######## Opatroides vicinus

Taxon classificationAnimaliaColeopteraCleridae

(Fairmaire, 1896)

######### World distribution.

**Asia**: AE, AF, BH, IN, IR, KW, NP, OM, PK, SA, YE.

######### General distribution.

ORR_SAR.

######### Local distribution.

RI (Kaszab 1979 1982).

######### Collecting month and method.

Abundant species. The specimens of this species were collected by BV on branches of *Acacia gerrardii* in IV; and by HP, LT and PT throughout the year except in IX and XI.

######## Palorus ficicola

Taxon classificationAnimaliaColeopteraCleridae

(Wollaston, 1867)

######### World distribution.

**Africa**: CV, GM. **Asia**: AE (Schawaller 2010), LK, PK, SA. **North Africa**: DZ, EG, LY, MA.

######### General distribution.

AFR_ORR_SAR.

######### Local distribution.

MK (Kaszab 1979 1982).

######### Collecting month and method.

Very rare species, which was collected by LT during VIII.

######## Praeugena gagatina

Taxon classificationAnimaliaColeopteraCleridae

(Mäklin, 1863)

######### World distribution.

**Africa**: DJ, ER, ET, SD, SN, SO, TD. **Asia**: AE (Schawaller 2010), SA, YE.

######### General distribution.

AFR_SAR.

######### Local distribution.

AS, JZ, MD, MK, RI (Kaszab 1979 1982).

######### Collecting month and method.

Rare species. The adults were collected by PT under canopy of *Lycium shawii* during V; and y LT in IV-V.

######## Prodilamus fausti major

Taxon classificationAnimaliaColeopteraCleridae

Kaszab, 1982

######### World distribution.

**Asia**: SA.

######### General distribution.

END.

######### Local distribution.

MD, QS (Kaszab 1982).

######### Collecting month and method.

Very rare species that was collected by HP during XII.

######## Sclerum carinatum

Taxon classificationAnimaliaColeopteraCleridae

Baudi 1875

######### World distribution.

**Asia**: AF, AZ, CY, IQ, IR, SA, SY, TJ, TM, TR, UZ.

######### General distribution.

PAL_SAR.

######### Local distribution.

RI (Kaszab 1979).

######### Collecting month and method.

Rare species that was collected by HP and LT during II-III, VII and IX.

######## Sclerum orientale

Taxon classificationAnimaliaColeopteraCleridae

(Fabricius, 1775)

######### World distribution.

**Africa**: SD. **Asia**: IL, JO, SA, SI, YE. **North Africa**: EG.

######### General distribution.

AFR_SAR.

######### Local distribution.

RI (Kaszab 1979 1982).

######### Collecting month and method.

A common species. The adult beetles were collected by BV on branches of *Acacia ehrenbergiana* during X; and by HP and PT all over the year except in VI and IX.

######## Sclerum sulcatum

Taxon classificationAnimaliaColeopteraCleridae

Baudi, 1876

######### World distribution.

**Asia**: SA. **North Africa**: EG.

######### General distribution.

SAR.

######### Local distribution.

HA, MK, RI (Kaszab 1979 1982; Johnson 1989).

######### Collecting month and method.

A frequent species. The specimens were collected by HP, LT and PT during I-VII, X and XII.

####### Subfamily: Alluculinae

######## Cornucistela serrata

Taxon classificationAnimaliaColeopteraCleridae

Campbell, 1980

######### World distribution.

**Asia**: SA.

######### General distribution.

END.

######### Local distribution.

RI (Campbell 1980).

######### Collecting month and method.

Rare species that was collected by LT during V-VI and VIII-X.

######## Cteniopus pallidus

Taxon classificationAnimaliaColeopteraCleridae

(Küster, 1850)

######### World distribution.

**Asia**: CY, IQ, SY, TR. New to Arabian Peninsula.

######### General distribution.

SAR.

######### Collecting month and method.

Very rare species that was collected by SW during IV.

######## Hymenalia denticulata

Taxon classificationAnimaliaColeopteraCleridae

(Muche, 1982)

######### World distribution.

**Asia**: AE, OM, SA.

######### General distribution.

SAR.

######### Local distribution.

AS, BA, MD, MK (Muche 1982).

######### Collecting month and method.

Frequent species. The adults were collected by LT during IV-VI, IX and XI.

######## Mycetocharina bahukalatensis

Taxon classificationAnimaliaColeopteraCleridae

Novak, 2008

######### World distribution.

**Asia**: IR. New to Arabian Peninsula

######### General distribution.

SAR.

######### Collecting month and method.

A frequent species that was collected by LT in IV-X.

######## Mycetocharina braaschi

Taxon classificationAnimaliaColeopteraCleridae

Muche, 1982

######### World distribution.

**Asia**: AE, SA.

######### General distribution.

SAR.

######### Local distribution.

MK, RI (Muche 1982).

######### Collecting month and method.

Common species. The specimens were collected by LT in IV-VI and X.

###### Oedemeridae

####### Subfamily: Oedemerinae

######## Alloxantha talhouki

Taxon classificationAnimaliaColeopteraCleridae

Švihla, 1984

######### World distribution.

**Asia**: AE, IR, OM, SA.

######### General distribution.

SAR.

######### Local distribution.

EP (Švihla 1984).

######### Collecting month and method.

Very rare species. The specimens were collected by LT during V-VI.

###### Meloidae

####### Subfamily: Meloinae

######## Lydomorphus angusticollis suturellus

Taxon classificationAnimaliaColeopteraCleridae

(Haag-Rutenberg, 1880)

######### World distribution.

**Asia**: AE, IR, OM, PK, SA, YE.

######### General distribution.

SAR.

######### Local distribution.

EP, MD, MK, RI, (Kaszab 1983, Schneider 1991).

######### Collecting month and method.

Very rare species that was collected by LT through IV.

######## Lydomorphus brittoni

Taxon classificationAnimaliaColeopteraCleridae

(Kaszab 1953)

######### World distribution.

**Asia**: AE, IQ, OM, SA.

######### General distribution.

SAR.

######### Local distribution.

EP, MD, MK, QS, RI, (Kaszab 1983, Schneider 1991).

######### Collecting month and method.

Frequent species, which was collected by LT during IV.

######## Lydomorphus palaestinus

Taxon classificationAnimaliaColeopteraCleridae

(Kirsch, 1871)

######### World distribution.

**Asia**: EG (Sinai), IL, JO, SA. **North Africa**: DZ, EG, LY, MA, TN.

######### General distribution.

SAR.

######### Local distribution.

AS, EP, RI (Kaszab 1983, Schneider 1991).

######### Collecting month and method.

Very rare species that was collected by LT during III.

####### Subfamily: Nemognathinae

######## Zonitoschema rubricolor

Taxon classificationAnimaliaColeopteraCleridae

Pic, 1924

######### World distribution.

**Africa**: CD. **Asia**: AE (Batelka and Geisthardt 2009), IL, SA.

######### General distribution.

AFR_SAR.

######### Local distribution.

BA, RI (Kaszab 1983, Schneider 1991; El-Hawagry et al. 2013).

######### Collecting month and method.

Very rare species. The adults were collected by LT in VI.

###### Anthicidae

####### Subfamily: Anthicinae

######## Anthelephila caeruleipennis

Taxon classificationAnimaliaColeopteraCleridae

(Laferte-Senectere, 1847)

######### World distribution.

**Africa**: ET, ZA. **Asia**: AE, EG (Sinai), IL, IQ, IR, JO, LB, OM, PK, SA, SY, YE. **Europe**: ES, IT. **North Africa**: DZ, EG, ES (Canary Islands), LY, MA, TN.

######### General distribution.

AFR_SAR.

######### Local distribution.

AS, BA, JZ, MD, MK, QS (Uhmann 1998; El-Hawagry et al. 2013).

######### Collecting month and method.

Very rare species that was collected by LT during IV and VI.

######## Anthelephila multiformis

Taxon classificationAnimaliaColeopteraCleridae

Kejval, 2002

######### World distribution.

**Asia**: AE (Telnov 2007), IR, OM, PK. New to KSA.

######### General distribution.

SAR.

######### Collecting month and method.

Rare species. The specimens of this species were collected by BV, SW, VC and PT on branches/under canopies of *Acacia ehrenbergiana*, *Acacia gerrardii*, *Calotropis procera*, *Lycium shawii*, *Rhazya stricta* and *Ziziphus nummularia*; and by LT through IV-VI.

######## Anthicus crinitus

Taxon classificationAnimaliaColeopteraCleridae

Laferte-Senectere, 1849

######### World distribution.

**Africa**: CF, GM, KE, MR, NA, SL, SN, SZ, TD, ZA. **Asia**: AE, AF, AZ, CN, CY, EG (Sinai), IL, IN, IQ, IR, JO, JP, KW, NP, OM, PK, SA, SY, TH, TR, TW, UZ, YE. **Europe**: AL, AM, BG, GR, MT, PT, RU. **North Africa**: DZ, EG, ES (Canary Islands), LY, MA, PT (Madeira Archipelago), TN. **North America**: CU, DO, PR. **South America**: VE.

######### General distribution.

COS.

######### Local distribution.

AS, MK, QS, (Uhmann 1998), BA (El-Hawagry et al. 2013), RI (Al Dhafer et al. 2016).

######### Collecting month and method.

Rare species. The adults were collected by PT under canopies of *Acacia ehrenbergiana*; and by LT during V-VII and IX-X.

######## Anthicus tristis

Taxon classificationAnimaliaColeopteraCleridae

Schmidt, 1842

######### World distribution.

**Asia**: AF, AZ, CY, IL, IQ, IR, JO, KZ, LB, SA, SY, TJ, TM, TR, UZ, YE. **Europe**: AM, BG, ES, FR, GE, GR, HR, IT, MT, RO, RU, SE, SK, UA. **North Africa**: DZ, EG, ES (Canary Islands), LY, MA, TN.

######### General distribution.

PAL_SAR.

######### Local distribution.

RI (Uhmann 1992).

######### Collecting month and method.

Very rare species that was collected by LT in IX.

######## Endomia lefebvrei

Taxon classificationAnimaliaColeopteraCleridae

(LaFerté-Sénectěre, 1849)

######### World distribution.

**Africa**: GM, TD. **Asia**: AE, AF, CY, EG (Sinai), IL, IQ, IR, JO, OM, QA, SA, TM, TR, YE. **North Africa**: DZ, EG, LY, MA, TN.

######### General distribution.

AFR_PAL_SAR.

######### Local distribution.

BA (El-Hawagry et al. 2013), JZ, MK (Uhmann 1998), RI (Al Dhafer et al. 2016).

######### Collecting month and method.

A rare species. The beetles were collected by PT under canopies of *Acacia gerrardii*, *Calotropis procera* and *Rhazya stricta*; and by LT during IV-VI.

######## Omonadus floralis

Taxon classificationAnimaliaColeopteraCleridae

(Linnaeus, 1758)

######### World distribution.

**Africa**: CM, MR, SN, ZA. **Asia**: AE (Telnov 2007), AF, AZ, BT, CN, CY, EG (Sinai), IL, IN, IR, JO, JP, LB, MN, NP, OM, PK, RU, SA, SY, TM, TR. **Australia**: PG. **Europe**: AL, AR, AT, BA, BE, BG, BY, CH, DE, DK, EE, ES, FI, FR, GB, GE, GR, HR, HU, IE, IT, LI, LT, LV, MD, MK, MT, NL, NO, PL, PT, RO, RS, RU, SE, SK, TR, UA. **North Africa**: DZ, EG, ES (Canary Islands), LY, MA, PT (Madeira Archipelago), TN. **North America**: CA, US.

######### General distribution.

COS (Uhmann 1998, Telnov 2007).

######### Local distribution.

BA, MK (Uhmann 1998), RI (Alqarni et al. 2015; Al Dhafer et al. 2016).

######### Collecting month and method.

Very rare species that was collected by PT under canopies of *Ziziphus nummularia*, and by LT during V-VI.

####### Subfamily: Notoxinae

######## Mecynotarsus bison

Taxon classificationAnimaliaColeopteraCleridae

(Olivier, 1811)

######### World distribution.

**Africa**: CG, CI, CV, ET, SD, SO, TD, TZ. **Asia**: AE, CY, EG (Sinai), IL, IQ, IR, LB, SA, TR, YE. **Europe**: GR. **North Africa**: DZ, EG, ES (Canary Islands), LY, MA, TN.

######### General distribution.

AFR_PAL_SAR.

######### Local distribution.

EP, JZ, MD, MK, NB, RI, TB (Uhmann 1998).

######### Collecting month and method.

Very rare species that was collected by PT under canopy of *Rhazya stricta* in IV.

###### Scraptiidae

####### Subfamily: Anaspidinae

######## Pentaria arabica

Taxon classificationAnimaliaColeopteraCleridae

Pankow, 1981

######### World distribution.

**Asia**: SA.

######### General distribution.

END.

######### Local distribution.

AS (Pankow 1981).

######### Collecting month and method.

A frequent species. It was collected by BV and PT on branches/under canopy of *Acacia ehrenbergiana*, and by LT and MT through IV-V, VII and X.

######## Pentaria sp.

Taxon classificationAnimaliaColeopteraCleridae

######### Collecting month and method.

Rare species. The specimens were collected by BV on branches of *Ziziphus nummularia* in V, by PT under canopy of *Lycium shawii* in V, and by LT during V-VII and IX.

###### Cerambycidae

####### Subfamily: Prioninae

######## Polyarthron philbyi

Taxon classificationAnimaliaColeopteraCleridae

Villiers, 1968

######### World distribution.

**Asia**: SA.

######### General distribution.

END.

######### Local distribution.

RI (Holzschuh 1993).

######### Collecting month and method.

Very rare species. It was collected by HP during IX.

####### Subfamily: Lamiinae

######## Apomecyna lameerei

Taxon classificationAnimaliaColeopteraCleridae

(Pic, 1895)

######### World distribution.

**Africa**: MR. **Asia**: AE, IL, IQ, IR, PK, SA. **North Africa**: EG.

######### General distribution.

AFR_SAR.

######### Local distribution.

MD, MK, RI (Holzschuh and Téocchi 1991).

######### Collecting month and method.

Rare species. The adults were collected by HP and LT during IV-V, X-XI.

###### Chrysomelidae

####### Subfamily: Bruchinae

######## Bruchidius centromaculatus

Taxon classificationAnimaliaColeopteraCleridae

(Allard, 1868)

######### World distribution.

**Africa**: BF, CD, MR, SD, SN. **Asia**: IL, SA, YE. **North Africa**: EG.

######### General distribution.

AFR_SAR.

######### Local distribution.

AS (Anton 1994), RI (Al Dhafer et al. 2016).

######### Collecting month and method.

Common species, which was collected by BV, SW and VC on branches of *Acacia ehrenbergiana*, *Acacia gerrardii*, *Calotropis procera*, *Lycium shawii*, *Rhazya stricta* and *Ziziphus nummularia*, and by HP, LT and PT throughout the year.

######## Careydon acaciae

Taxon classificationAnimaliaColeopteraCleridae

(Gyllenhall, 1833)

######### World distribution.

**Africa**: AO, BF, CD, CM. ET, KE, MR, MZ, NA, NE, NG, RW, SD, SN, SO, SZ, TD, TZ, ZA. **Asia**: IR, JO, SA, YE. **Europe**: ES, HU. **North Africa**: EG.

######### General distribution.

AFR_SAR.

######### Local distribution.

AS, MK (Decelle 1979), RI (Al Dhafer et al. 2016)

######### Collecting month and method.

Common species. The specimens were collected by PT under the canopies of *Acacia ehrenbergiana*, *Acacia gerrardii*, *Calotropis procera* and *Lycium shawii*; and by SW on branches of *Calotropis procera* and *Rhazya stricta*; and by HP, LT and MT throughout the year.

######## Spermophagus sericeus

Taxon classificationAnimaliaColeopteraCleridae

(Geoffory, 1785)

######### World distribution.

**Asia**: AF, AZ, CN, CY, IL, IQ, IR, JO, KG, KZ, LB, MN, SA, SY, TJ, TM, TR, UZ. **Europe**: AL, AM, AT, BA, BE, BG, CH, CZ, DE, DK, ES, FI, FR, GB, GE, GR, HR, HU, IT, LU, MK, MT, NL, PL, PT, RO, RS, RU, SE, SI, SK, UA. **North Africa**: DZ, MA, TN.

######### General distribution.

PAL_SAR_SJP.

######### Local distribution.

RI (Anton 1994).

######### Collecting month and method.

Very rare that species was collected by SW on branches of *Calotropis procera* during VIII.

####### Subfamily: Chrysomelinae

######## Colaphellus apicalis

Taxon classificationAnimaliaColeopteraCleridae

(Ménétriés, 1849)

######### World distribution.

**Asia**: AF, IL, IR, KG, KZ, SY, TM, TR, UZ. New to Arabian Peninsula.

######### General distribution.

PAL_SAR.

######### Collecting month and method.

Very rare species. It was collected by PT under the canopy of *Rhazya stricta* during II.

####### Subfamily: Galerucinae

######## Phyllotreta lativittata

Taxon classificationAnimaliaColeopteraCleridae

Kutschera, 1860

######### World distribution.

**Asia**: AF, AZ, CN, CY, IL, IQ, IR, JO, KG, KZ. LB, OM, RU, SA (Al Dhafer et al. 2016), SY, TJ, TR, TM, UZ. **Europe**: AM, GR, IT, MT.

######### General distribution.

PAL_SAR_SJP.

######### Local distribution.

RI (Al Dhafer et al. 2016).

######### Collecting month and method.

Common species. The adult beetles were collected by BV, SW, VC and PT on branches/under the canopies of *Acacia ehrenbergiana*, *Acacia gerrardii*, *Calotropis procera*, *Lycium shawii*. *Rhazya stricta* and *Ziziphus nummularia*; and by LT and MT throughout the year.

######## Psylliodes peyerimhoffi

Taxon classificationAnimaliaColeopteraCleridae

Heikertinger, 1916

######### World distribution.

**Asia**: AE, EG (Sinai), SA.

######### General distribution.

SAR.

######### Local distribution.

RI (Doguet 1979; Medvedev 1996).

######### Collecting month and method.

Very rare species that was collected by LT during IV.

####### Subfamily: Cryptocephalinae

######## Aetheomorpha seminigra pumilio

Taxon classificationAnimaliaColeopteraCleridae

(Lacordaire 1848)

######### World distribution.

**Africa**: ET, SD. **Asia**: AF, JO, OM, SA, YE. **North Africa**: EG.

######### General distribution.

AFR_SAR.

######### Local distribution.

AS, BA, JZ, MK (Medvedev 1993 1996; El-Hawagry et al. 2013).

######### Collecting month and method.

Very rare species, which was collected by BV on branches of *Acacia gerrardii* during V.

####### Subfamily: Eumolpinae

######## Macrocoma lefevrei

Taxon classificationAnimaliaColeopteraCleridae

(Baly, 1878)

######### World distribution.

**Asia**: IR, OM, SA. **North Africa**: EG.

######### General distribution.

SAR.

######### Local distribution.

MK (Medvedev 1996).

######### Collecting month and method.

Very rare species. It was collected by LT during IV.

###### Brentidae

####### Subfamily: Apioninae

######## Aplemonus arabicus

Taxon classificationAnimaliaColeopteraCleridae

(Wagner, 1909)

######### World distribution.

**Africa**: MW, ZA. **Asia**: AE (Magnano et al. 2009), IR, OM, QA (Magnano et al. 2009), SA (Al Dhafer et al. 2016). **North Africa**: EG.

######### General distribution.

AFR_SAR.

######### Local distribution.

RI (Al Dhafer et al. 2016)

######### Collecting month and method.

Common species. The beetles of this species were collected by BV, SW and VC on branches of *Acacia ehrenbergiana*, *Acacia gerrardii*, *Lycium shawii* and *Ziziphus nummularia*; by PT under canopies of *Calotropis procera*; and by LT throughout the year.

####### Subfamily: Nanophyinae

######## Allomalia quadrivirgata

Taxon classificationAnimaliaColeopteraCleridae

(A. Costa, 1863)

######### World distribution.

**Asia**: AZ, CY, IL, KZ, SA (Abdel-Dayem et al. 2015), SY, UZ. **Europe**: BG, ES, FR, GE, GR, HR, HU, IT, UA. **North Africa**: DZ, EG, LY, MA, TN.

######### General distribution.

PAL_SAR.

######### Local distribution.

RI (Abdel-Dayem et al. 2015).

######### Collecting month and method.

Very rare species that was collected by BV on branches of *Ziziphus nummularia* in IV.

###### Curculionidae

####### Subfamily: Curculioninae

######## Assuanensius discoidalis

Taxon classificationAnimaliaColeopteraCleridae

(Tournier, 1873)

######### World distribution.

**Africa**: TD. **Asia**: IL, SA (Abdel-Dayem et al. 2015). **North Africa**: DZ, EG, LY, MA.

######### General distribution.

AFR_SAR.

######### Local distribution.

RI (Abdel-Dayem et al. 2015).

######### Collecting month and method.

Common species. The specimens were collected by BV on branches of *Acacia ehrenbergiana* in III-V.

######## Assuanensius erectesetosus

Taxon classificationAnimaliaColeopteraCleridae

(Peyerimhoff, 1948)

######### World distribution.

**Africa**: TD. **Asia**: IL, SA (Abdel-Dayem et al. 2015). **North Africa**: EG, LY, MA.

######### General distribution.

AFR_SAR.

######### Local distribution.

RI (Abdel-Dayem et al. 2015).

######### Collecting month and method.

Very rare species. The beetles were collected by BV on branches of *Acacia ehrenbergiana*, *Acacia gerrardii*, *Calotropis procera*, *Lycium shawii*, *Rhazya stricta* and *Ziziphus nummularia* during I-VII and IX; by PT under canopies of *Calotropis procera* and *Rhazya stricta* in III; and by HP and SW in II and III respectivelly.

######## Assuanensius peyerimhoffi

Taxon classificationAnimaliaColeopteraCleridae

(Hoffmann, 1963)

######### World distribution.

**Africa**: TD. **Asia**: SA (Abdel-Dayem et al. 2015). **North Africa**: DZ, LY.

######### General distribution.

AFR_SAR.

######### Local distribution.

RI (Abdel-Dayem et al. 2015).

######### Collecting month and method.

A frequent species. The specimens were collected by BV on branches of *Acacia ehrenbergiana*, *Acacia gerrardii* and *Ziziphus nummularia* during I-III, V, VIII, and IX-XII.

######## Mecinus longulus

Taxon classificationAnimaliaColeopteraCleridae

(Desbrochers des Loges, 1893)

######### World distribution.

**Asia**: IL, SA (Abdel-Dayem et al. 2015). **North Africa**: DZ, EG, LY, TN.

######### General distribution.

SAR.

######### Local distribution.

RI (Abdel-Dayem et al. 2015).

######### Collecting month and method.

Very rare species that was collected by BV on branches of *Lycium shawii* and by PT under canopy of *Rhazya stricta* during III.

######## Pachytychius cognatus

Taxon classificationAnimaliaColeopteraCleridae

Caldara, 2000

######### World distribution.

**Africa**: AO, DJ, SD, SN. **Asia**: SA, YE.

######### General distribution.

AFR_SAR.

######### Local distribution.

JZ (Caldara 2000).

######### Collecting month and method.

Very rare species, which was collected by PT under canopy of *Rhazya stricta* in XII.

######## Pseudorchestes letourneuxi

Taxon classificationAnimaliaColeopteraCleridae

(Pic, 1901)

######### World distribution.

**Africa**: SD. **Asia**: SA (Abdel-Dayem et al. 2015), TR. **North Africa**: DZ, EG, LY, MA, TN.

######### General distribution.

AFR_SAR.

######### Local distribution.

RI (Abdel-Dayem et al. 2015).

######### Collecting month and method.

Very rare species that was collected by PT under canopy of *Ziziphus nummularia* during IV.

######## Sharpia sabulicola

Taxon classificationAnimaliaColeopteraCleridae

Colonnelli, 2009

######### World distribution.

**Asia**: AE (Magnano et al. 2009), SA (Abdel-Dayem et al. 2015).

######### General distribution.

SAR.

######### Local distribution.

RI (Abdel-Dayem et al. 2015).

######### Collecting month and method.

Very rare species and its adult was collected by LT during IV.

######## Sharpia soluta

Taxon classificationAnimaliaColeopteraCleridae

Faust, 1885

######### World distribution.

**Asia**: AE (Magnano et al. 2009), AZ, CY, SA (Abdel-Dayem et al. 2015), TM, UZ. **Europe**: RO.

######### General distribution.

PAL_SAR.

######### Local distribution.

RI (Abdel-Dayem et al. 2015).

######### Collecting month and method.

Rare species. It was collected by PT under canopies of *Acacia ehrenbergiana*, *Calotropis procera*, *Rhazya stricta*, and *Ziziphus nummularia* during III-IV, X and XII; also collected by LT during V.

######## Sphincticraerus bruleriei

Taxon classificationAnimaliaColeopteraCleridae

(Desbrochers des Loges, 1873)

######### World distribution.

**Asia**: IL, SA (Abdel-Dayem et al. 2015). **North Africa**: EG.

######### General distribution.

SAR.

######### Local distribution.

RI (Abdel-Dayem et al. 2015).

######### Collecting month and method.

A rare species. The adult beetles were collected by BV on branches of *Ziziphus nummularia* during II and V; and by LT through X.

######## Tychius banfii

Taxon classificationAnimaliaColeopteraCleridae

Caldara & Fremuth, 1992

######### World distribution.

**Asia**: IQ, IR, JO, SA, TR. **North Africa**: EG.

######### General distribution.

PAL_SAR.

######### Local distribution.

EP, RI (Caldara 1993).

######### Collecting month and method.

Rare species. Its adult was collected by BV on branches of *Acacia ehrenbergiana*, *Acacia gerrardii* and *Ziziphus nummularia* through XII; by PT under canopy of *Acacia ehrenbergiana* in XI; and by LT and MT during V and VII respectivelly.

######## Tychius mozabitus

Taxon classificationAnimaliaColeopteraCleridae

Pic, 1898

######### World distribution.

**Asia**: EG (Sinai), IQ, IR, JO, SA. **North Africa**: DZ, EG, LY, TN.

######### General distribution.

SAR.

######### Local distribution.

MD, RI (Caldara 1993).

######### Collecting month and method.

Very rare species that was collected by PT under canopy of *Rhazya procers* in II.

######## Tychius vicinus

Taxon classificationAnimaliaColeopteraCleridae

Roudier, 1954

######### World distribution.

**Asia**: AE (Magnano et al. 2009), SA, YE. **North Africa**: DZ, EG.

######### General distribution.

SAR.

######### Local distribution.

AS, RI (Caldara 1993).

######### Collecting month and method.

A frequent species. The adults were collected by BV, SW and VC on branches of *Acacia gerrardii*, *Rhazya stricta* and *Ziziphus nummularia* during III-V; and by PT under canopy of *Acacia ehrenbergiana* during I-VI and XII.

####### Subfamily: Entiminae

######## Myllocerus sp.

Taxon classificationAnimaliaColeopteraCleridae

######### Collecting month and method.

A rare species. It was collected by PT under canopies of *Rhazya stricta* and *Ziziphus nummularia* during I-II; and by HP and LT in I, IV and XII.

######## Tanymecus musculus

Taxon classificationAnimaliaColeopteraCleridae

Fåhraeus, 1840

######### World distribution.

**Asia**: IL, IQ, SA (Abdel-Dayem et al. 2015), SY. **North Africa**: EG.

######### General distribution.

SAR.

######### Local distribution.

RI (Abdel-Dayem et al. 2015).

######### Collecting month and method.

Very rare species that was collected by LT in I, IV and XII.

####### Subfamily: Hyperinae

######## Brachypera isabellina

Taxon classificationAnimaliaColeopteraCleridae

(Boheman, 1834)

######### World distribution.

**Asia**: AE (Magnano et al. 2009), IL, IQ, IR, JO, KW, QA, SA, SY, TR. **Europe**: IT (Sicilia). **North Africa**: DZ, EG, ES (Canary Islands), LY, MA, PT (Madeira Archipelago), TN.

######### General distribution.

SAR.

######### Local distribution.

Heyden (1913) just mentioned Arabia without any further detail about locality. Abdel-Dayem et al. (2015) have been confirmed the occurrence of the species in KSA: RI.

######### Collecting month and method.

A rare species. The beetles were collected by PT under canopies of *Calotropis procera*, *Rhazya stricta* and *Ziziphus nummularia* during II, VIII and XII.

######## Hypera brunnipennis

Taxon classificationAnimaliaColeopteraCleridae

(Boheman, 1834)

######### World distribution.

**Asia**: IL, IR, LB, SA (Abdel-Dayem et al. 2015). **North Africa**: EG. **North America**: US.

######### General distribution.

NAR_SAR.

######### Local distribution.

RI (Abdel-Dayem et al. 2015).

######### Collecting month and method.

Very rare species that was collected by LT during IV.

####### Subfamily: Lixinae

######## Hypolixus pica

Taxon classificationAnimaliaColeopteraCleridae

(Fabricius, 1798)

######### World distribution.

**Africa**: CG, ET, NE, SN, TD, TG. **Asia**: AE, CY, ID, IQ, IR, JO, PK, SA (Abdel-Dayem et al. 2015), SY, TR, YE (Socotra). **Europe**: FR. **North Africa**: EG.

######### General distribution.

AFR_PAL_SAR.

######### Local distribution.

RI (Abdel-Dayem et al. 2015).

######### Collecting month and method.

Very rare species that was collected by BV on branches of *Acacia ehrenbergiana* in XI.

######## Larinus elegans

Taxon classificationAnimaliaColeopteraCleridae

Desbrochers des Loges, 1897

######### World distribution.

**Asia**: SA. **North Africa**: DZ, EG, LY, MA.

######### General distribution.

SAR.

######### Local distribution.

There is no available information.

######### Collecting month and method.

Very rare species. The adult of this species was collected by BV on branches of *Ziziphus nummularia* through V.

######## Lixus ? subfarinosus

Taxon classificationAnimaliaColeopteraCleridae

Desbrochers des Loges, 1893

######### World distribution.

**Asia**: AE, IQ, SA (Abdel-Dayem et al. 2015), TR, YE. **North Africa**: EG. TN.

######### General distribution.

SAR.

######### Local distribution.

RI (Abdel-Dayem et al. 2015).

######### Collecting month and method.

Very rare species that was collected by HP in IV.

######## Pycnodactylopsis tomentosa

Taxon classificationAnimaliaColeopteraCleridae

(Fåhraeus, 1842)

######### World distribution.

**Africa**: ET, MR, SD, TD. **Asia**: EG (Sinai), IL, IN, IQ, IR, JO, PK, SA, YE. **North Africa**: DZ, EG, ES (Canary Islands), LY, MA, TN.

######### General distribution.

AFR_ORR_SAR.

######### Local distribution.

RI (Shalaby 1961, Beccari 1971).

######### Collecting month and method.

Very rare species that was collected by HP in II.
